# Screening Mangrove Endophytic Fungi for Antimalarial Natural Products

**DOI:** 10.3390/md11125036

**Published:** 2013-12-12

**Authors:** Laurent Calcul, Carrie Waterman, Wai Sheung Ma, Matthew D. Lebar, Charles Harter, Tina Mutka, Lindsay Morton, Patrick Maignan, Alberto Van Olphen, Dennis E. Kyle, Lilian Vrijmoed, Ka-Lai Pang, Cedric Pearce, Bill J. Baker

**Affiliations:** 1 Department of Chemistry, University of South Florida, 4202 E. Fowler, CHE 205, Tampa, FL 33620, USA; E-Mails: calcul@usf.edu (L.C.); waterman@aesop.rutgers.edu (C.W.); wma2@mail.usf.edu (W.S.M.); mlebar@chemistry.harvard.edu (M.D.L.); csharter41@gmail.com (C.H.); 2 Department of Global Health, University of South Florida, 3720 Spectrum Blvd., Suite 304, Tampa, FL 33612, USA; E-Mails: tmutka@health.usf.edu (T.M.); wst8@cdc.gov (L.M.); pmaignan@mail.usf.edu (P.M.); avanolph@health.usf.edu (A.V.O.); dkyle@health.usf.edu (D.E.K.); 3 Department of Biology and Chemistry, City University of Hong Kong, 83 Tat Chee Avenue, Hong Kong, China; E-Mail: bhlilian@cityu.edu.hk; 4 Institute of Marine Biology and Center of Excellence for the Oceans, National Taiwan Ocean University, 2 Pei-Ning Road, Keelung 20224, Taiwan; E-Mail: klpang@ntou.edu.tw; 5 Mycosynthetix, Inc., 505 Meadowlands Drive, Suite 103, Hillsborough, NC 27278, USA; E-Mail: cpearce@mycosynthetix.com; 6 Center for Drug Discovery and Innovation, University of South Florida, 3720 Spectrum Blvd., Suite 303, Tampa, FL 33612, USA

**Keywords:** fungi, endophytes, mangroves, malaria, cytotoxicity, high-throughput

## Abstract

We conducted a screening campaign to investigate fungi as a source for new antimalarial compounds. A subset of our fungal collection comprising Chinese mangrove endophytes provided over 5000 lipophilic extracts. We developed an accelerated discovery program based on small-scale cultivation for crude extract screening and a high-throughput malaria assay. Criteria for hits were developed and high priority hits were subjected to scale-up cultivation. Extracts from large scale cultivation were fractionated and these fractions subjected to both *in vitro* malaria and cytotoxicity screening. Criteria for advancing fractions to purification were developed, including the introduction of a selectivity index and by dereplication of known metabolites. From the Chinese mangrove endophytes, four new compounds (**14**–**16**, **18**) were isolated including a new dimeric tetrahydroxanthone, dicerandrol D (**14**), which was found to display the most favorable bioactivity profile.

## 1. Introduction

Mangrove plants and their associated microfauna have been a rich source of bioactive molecules [1,2,3], though only limited [4,5,6] antimalarial screening of this chemodiversity source has been reported. Mangrove forests are fascinating and complex ecosystems [7]. They serve coastal populations worldwide by protecting shorelines from storm surge and erosion, through filtration/remediation of terrestrial runoff, and as nurseries for important fisheries, among other useful roles. Yet these marine margin communities are under constant threat of clearing from real estate development, fish-pond farming, and even for their wood to support cooking hearths in poor, rural communities. The World Wildlife Foundation [8] reports that 35% of global mangrove communities have disappeared in the last two decades alone [9,10], and one in six species are in danger of extinction [11,12]. Similar to the more visible coral reef and rainforest ecosystems, a better understanding of the biotechnological value of marine margin communities may engender respect and enthusiasm for conservation efforts. We report here a screening campaign using mangrove endophytic fungi from the Mai Po Nature reserve, Hong Kong, and Hainan Island, Taiwan, as sources for new antimalarial compounds.

## 2. Results and Discussion

### 2.1. Strategy

**Scheme 1:**
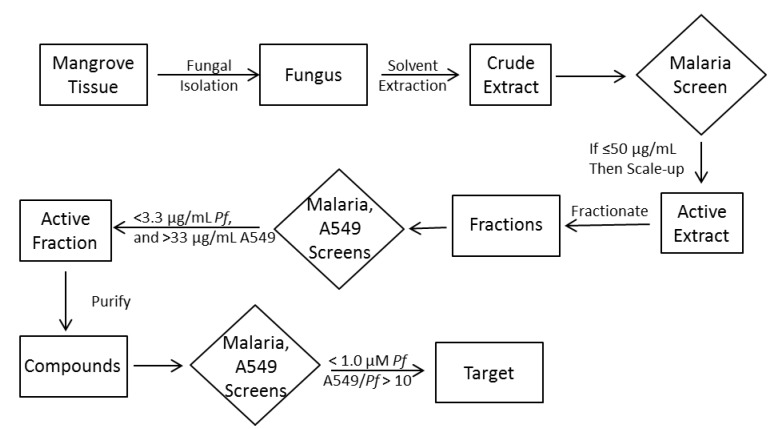
Sample workflow and decision points (*Pf* = *Plasmodium falciparum*).

Our strategy to miniaturize fungal cultures provided for an economy of scale that allowed us to rapidly produce small quantities of crude extracts for screening. Using a robust and validated *Plasmodium falciparum* (3D7, a drug sensitive strain) screen [13], 96-well plated crude extract screening data was available to us weekly. Our workflow (Scheme 1) included decision points based on crude extract activity, leading to scaled-up cultivation, then new decision points based both on IC_50_ values of parasite and mammalian (A549) cytotoxicity. All purified metabolites were then screened for bioactivity and characterized either by LC/MS dereplication with verification by NMR, or by de novo structure analysis. Over a two year period we cultured, screened and prioritized approximately 50,000 total fungal extracts, and conducted fractionation, re-screening, and purification on approximately 10% of those. Such an intensive effort requires trade-offs; we chose, for example, to conduct a single extract of freeze-dried cultures since it yielded sufficient material for screening, and we chose to forego verification of activity in scaled-up cultures, since fractionation was quick and fraction activity was more important than extract activity. Similarly, dereplication of known compounds was done late in the workflow. We reasoned that previously published compounds were still of interest to us, and even nuisance compounds might mask compounds of interest, so we chose to dereplicate only at the purification step (end) of the workflow. Such sharp focus on a bioactivity criterion (Scheme 1) left many potentially useful fractions along the workflow, fractions which will be deconvoluted in ensuing studies.

### 2.2. Fungal Isolation and Fermentation

Hong Kong and Taiwan have a similar subtropical climate and harbor many species of mangrove trees along the coastline where there is inundation of estuarine waters. *Kandelia obovata* and *Avicennia marina* are the dominant tree species in both healthy mangrove areas of Hong Kong and Taiwan while *Lumnitzera* *racemosa* is only found in Hong Kong mangrove communities. The endophytic fungal strains used in the study were isolated from surface sterilized plant tissues, using either 4% sodium hypochlorite [14] or 75% ethanol combined with 5% sodium hypochlorite solution [15]. Leaf and bark tissues of *A. marina*, *K. obovata* and *L. racemosa* were studied. A total of 5486 fungi were isolated and cultivated for screening.

### 2.3. Extraction, Plating and Screening of Miniaturized Cultures

Freeze dried fungal mycelia were extracted with 15 mL of dichloromethane/methanol (1:1) for 24 h then transferred to a 20 mL scintillation vials arranged in 8 × 12 arrays where they were air dried and taken up in DMSO to approximately 30 mg/mL. After transfer of 100 μL aliquots into 96-well plates, screening at two concentrations (5 and 50 μg/mL) using our previously published protocol [13] was conducted. Samples were prioritized as Active if they inhibited 3D7 by ≥67% at 5 µg/mL, and Partially Active if there was >67% inhibition at 50 µg/mL, leading to approximately 0.6% extracts categorized as Active, ~5% Partially Active, and more than 90% inactive. We advanced all Active extracts and 10% of the Partially Active into scale-up cultivation studies.

### 2.4. Chromatographic Separation, Screening and Structure Elucidation

Freeze-dried biomass from 2 L cultures were exhaustively extracted with dichloromethane/methanol (1:1) and applied to Combiflash^®^ MPLC cartridges based on the manufacturers recommendation of cartridge size to analyte mass. A linear gradient from hexane to ethyl acetate and then methanol was conducted, collecting ten to twelve fractions/extract. Fractions were concentrated and re-submitted for 3D7 screening, and cytotoxicity screening against A549 human lung adenocarcinoma epithelial cells followed. The cytotoxicity screen introduced a new decision point whereby fractions and purified compounds had to exceed a threshold selectivity index (A549 activity/3D7 activity) of 10. Fractions were advanced to HPLC if their 3D7 activity was <3.3 μg/mL and they met the selectivity index criterion, although priority was initially given to fractions with <1.1 μg/mL to focus on the most promising fractions first.

Purified compounds were first dereplicated using mass and ^1^H NMR data searched against AntiBase and SciFinder databases. For new compounds, full spectroscopic data sets (^1^H, ^13^C, DEPT, COSY, HSQC, HMBC, NOESY, HRMS) were acquired and analyzed to arrive at structural assignments.

### 2.5. Fungal Chemistry and its Bioactivity

**Figure 1:**
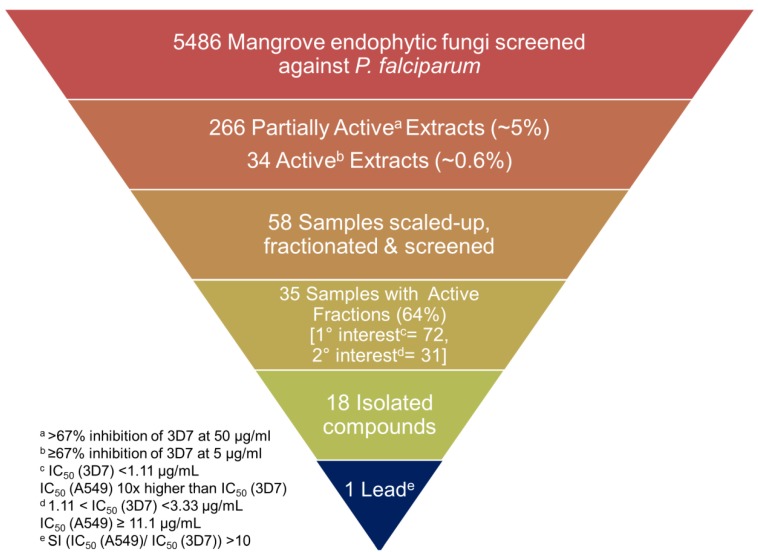
Summary of the screening results.

Of the 5486 fungi tested against *P. falciparum*, 266 were Partially Active (~5%) and 34 were Active (~0.6%). Of these, 58 samples were scaled-up and subjected to MPLC fractionation. A total of 103 MPLC fractions were identified for purification, and 18 compounds identified either through dereplication or structure analysis (Figure 1).

#### 2.5.1. Mycotoxins

**Figure 2:**
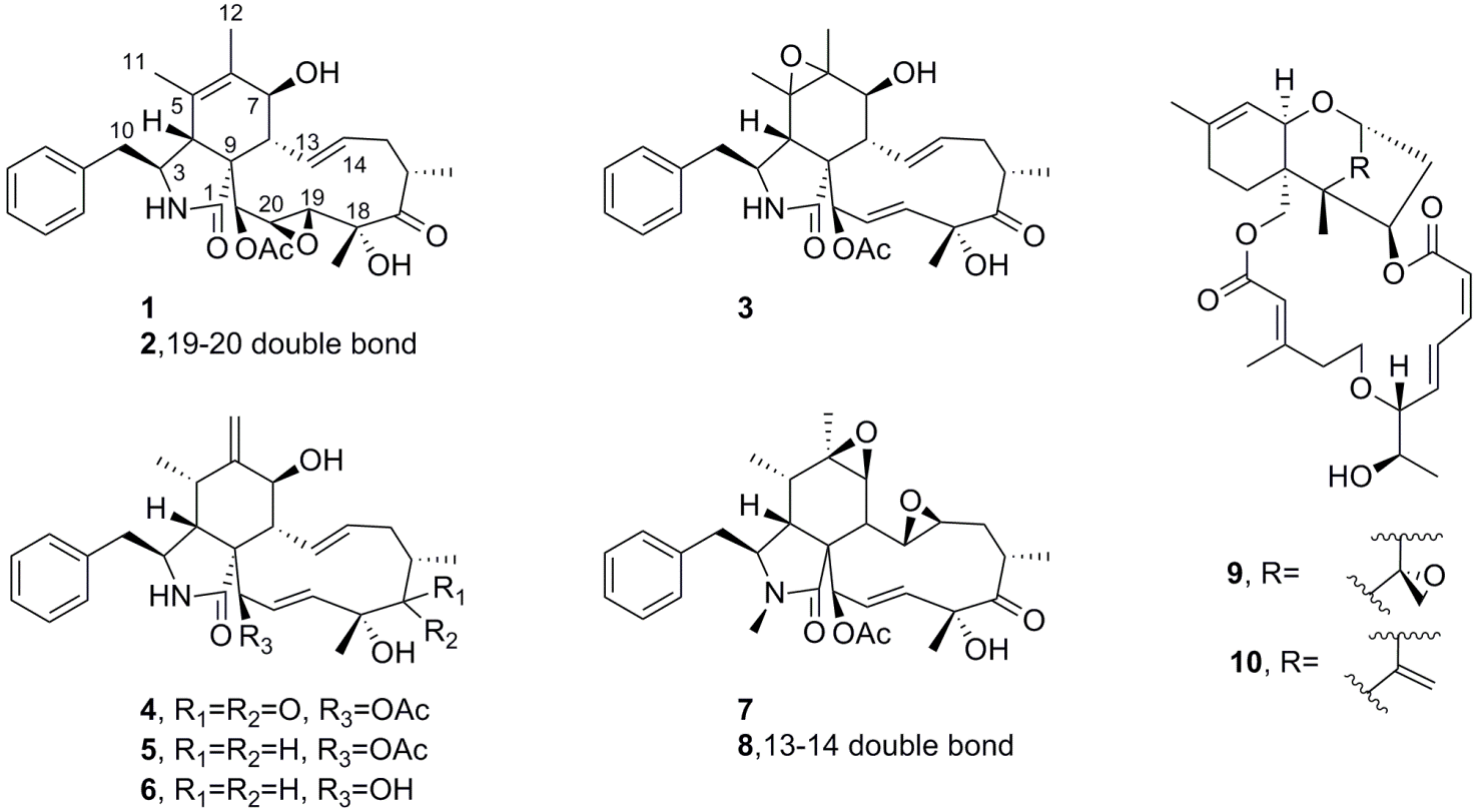
Mycotoxins from mangrove endophytes.

Mycotoxins proved to be the nuisance compounds in this study. Eight cytochalasins (**1**–**8**) and two trichothecenes (**9**, **10**) were dereplicated (Figure 2) from eight different fungal strains (Table 1), though others were not pursued to purity. All structures reported here bore spectroscopic data (ESIMS, ^1^H NMR) that agreed well with published [16,17,18,19,20] data. While often displaying potent antimalarial activity (Table 1), cytostatic activity that was not apparent in cytotoxicity screening precludes use of these toxins as drugs. Nonetheless, and in support of our decision to dereplicate late in the workflow, one trichothecene from another source remains one of the most promising leads of the project (work in progress).

**Table 1:** Compound summary and the associated anti-malarial activity against *Plasmodium falciparum* (3D7) and cytotoxicity against A549 cells.

Group	Cpd	IC_50_ (nM)		S.I.	Source (Taxonomic Identification)
		3D7	A549	A549/3D7	
**Mycotoxin**					
Cytochalasin	**1**	<20	ND *	−	CY-5286 (*Diaporthe* sp.)
	**2**	136	ND *	−	CY-5331 (*Xylaria* sp.)
	**3**	<20	ND *	−	CY-5368 (*Verticillium* sp.)
	**4**	25.8	ND *	−	CY-5286 (*Diaporthe* sp.), CY-6884 (*Xylaria* sp.), NTOU-3332 (*Xylaria* sp.), NTOU-1430 (*Xylaria* sp.)
	**5**	<20	ND *	−	CY-5286 (*Diaporthe* sp.)
	**6**	<20	ND *	−	CY-5286 (*Diaporthe* sp.)
	**7**	290	ND *	−	CY-5331 (*Xylaria* sp.)
	**8**	26	ND *	−	CY-5331 (*Xylaria* sp.)
Trichothecene	**9**	<20	<200	−	CY-3923 ^†^
	**10**	<20	<200	−	CY-3923 ^†^
**Polyketide**	**11**	36,000	>38,700	−	NTOU-1455 (*Xylaria* sp.)
	**12**	>48,500	>48,500	−	NTOU-2009 (*Phomopsis* sp.)
	**13**	>25,000	>25,000	−	CY-5188 (*Diaporthe* sp.)
	**14**	600	7800	13	CY-5188 (*Diaporthe* sp.)
	**15**	>25,000	>25,000	−	CY-5286 (*Diaporthe* sp.)
	**16**	>25,000	>25,000	−	CY-5286 (*Diaporthe* sp.)
**Lipid**	**18**	>25,000	>25,000	−	CY-5331(*Xylaria* sp.)
	**19**	>25,000	>25,000	−	CY-6884 (*Xylaria* sp.)
**Control**	**CQ**	4.5	>25,000	−	
	**ATO**	0.3	>25,000	−	
	**DHA**	0.8	>25,000	−	

S.I., selectivity index; * cytotostatic (recorded as lack of cell proliferation without cell death); ^†^ Not identified; ND, not determined; CQ, chloroquine; ATO, atovaquone; DHA, dihydroartemisinin.

#### 2.5.2. Polyketides

**Figure 3:**
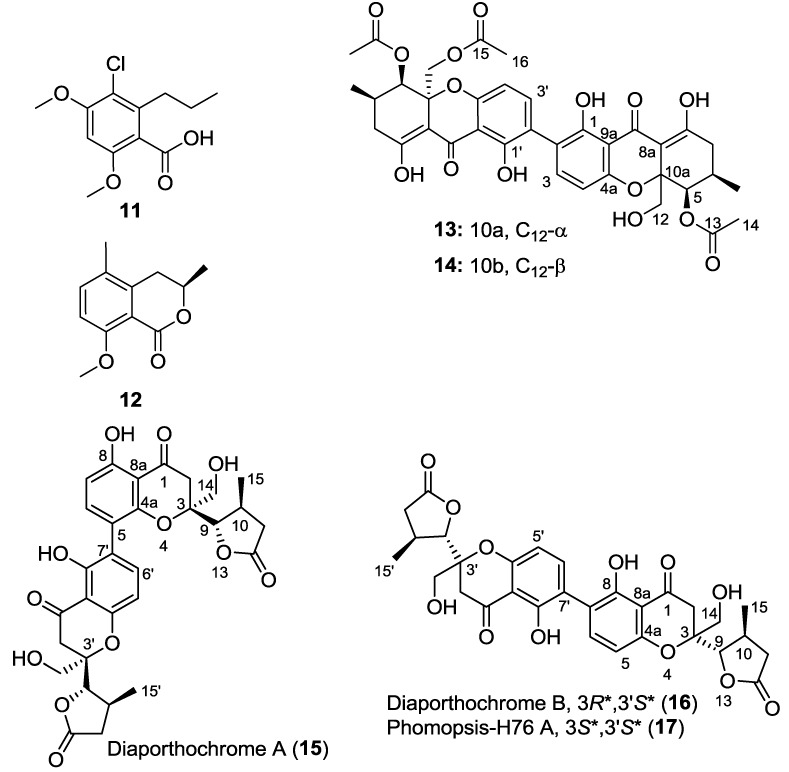
Polyketide chemodiversity from Chinese mangrove endophytes.

Six polyketides were found in our Chinese endophytic fungi (Figure 3). Two previously reported isolates, acremonisol A (**11**) [21] and 3,5-dimethyl-8-methoxy-3,4-dihydroisocoumarin (**12**) [22,23], were devoid of antimalarial activity but derived from fractions found active due to the presence of cytochalasin D (**4**). Dicerandrol B (**13**) and a previously unreported derivative, named here as dicerandrol D (**14**), were found from a strain of *Diaporthe* sp. (CY-5188). Further work on a related active extract produced two further unreported compounds, **15** and **16** [24], from another strain of *Diaporthe* (CY-5286). Dicerandrol D (**14**) displayed nanomolar antimalarial activity and a low cytotoxicity with a selectivity index of 13 (Table 1).

Isomers **13** and **14** shared a molecular formula, C_36_H_37_O_15_ (HRESIMS *m/z* at [M + H]^+^: 709.2123 for **13**: 709.2137 for **14**; calc’d 709.2132). Isolated as bright yellow solids, **13** was dereplicated as the dimer dicerandrol B [25]. Isomer **14** displayed similar chemical shifts in the ^1^H NMR spectrum with dicerandrol B, and detailed analysis of gHMBC data failed to find a connectivity difference. However, the 2D ROESY spectrum tellingly found no correlation between H-5 (δ 5.82) and hydroxymethyl H_2_-12 (δ 5.02 and 3.39), leading us to assign a *trans* relationship between H-5 the hydroxymethine group on C-10a. Optical rotation of the two isomers are divergent, supporting their stereochemical isomerism (**14**: [α]^20^_D_ +12.6; **13**: [α]^20^_D_ −8.3 (our isolate, in CHCl_3_), [α]^25^_D_ −6.5, reported in CHCl_3_ for dicerandrol B [25]). Interestingly the epimer **14** showed strong activity against *P. falciparum* with only moderate cytotoxicity, whereas dicerandrol B (**13**) failed to show either activity (Table 1). While the modest structural difference is difficult to rationalize as leading to the observed bioactivities, the change in sign of the optical rotation may suggest sufficient atropisomerism to make **14** a better receptor binder than **13**.

**Figure 4:**
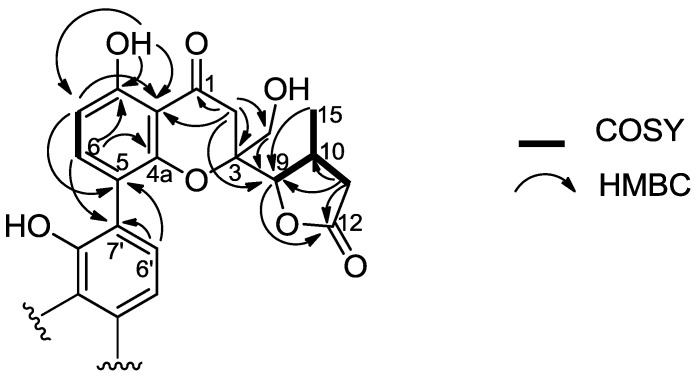
Selected HMBC and COSY correlations of **15**.

Compound **15** was obtained from strain CY-5286 which was identified as *Diaporthe* sp., the same genus as that yielding dicerandrols. Like **13** and **14**, compound **15** displayed a series of paired resonances in the ^13^C NMR spectrum, although only 30 resonances were observed (Table 2). The molecular formula of C_30_H_30_O_12_ was determined by HRESIMS ([M + H]^+^ 583.1842; [M + Na]^+^ 605.1630; calc’d for C_30_H_31_O_12_ 583.1816; calc’d for C_30_H_30_NaO_12_ 605.1635). However, **15** appeared to be a chromone, rather than xanthone, based on the presence of phenolic protons (δ_H_ 12.28 (8′-OH), 11.73 (8-OH)), but lack of enolic protons, among other differences. It also differed from **13** and **14** in that it lacked acetyl groups. The ^1^H NMR spectrum of **15** displayed aromatic signals on each of two monomeric units (δ_H-6_ 7.25 and δ_H-7_ 6.59; δ_H-5′_ 6.50 and δ_H-6′_ 7.29), which correlated in the COSY spectrum and could be assigned as *ortho* disposed based on the 8.8 and 8.3 Hz coupling constant, respectively. Focusing on one monomeric unit at a time, H-6 showed HMBC correlation to two aromatic carbons bearing oxygen (δ_C-4a_ 155.6 and δ_C-8_ 161.7), the latter of which proved to be a phenolic oxygen based on HMBC coupling from the δ_H_ 11.73 phenolic proton to δ_C-8_ 161.7. The C-8 phenolic proton showed further HMBC correlation to C-7 (δ_C_ 109.9) and C-8a (δ_C_ 107.0), and H-7 (δ_H_ 6.59) correlated with C-8a and C-5 (δ_C_ 115.2). That aromatic ring partial structure (Figure 4) was further developed by observation of HMBC correlations of H-2a (δ_H_ 3.22) to C-8a, the ketone carbon at C-1 (δ_C_ 196.9), an oxygenated quaternary carbon (δ_C-3_ 83.5), and with an oxygenated methine (δ_C-9_ 86.9). That H-9 (δ_H_ 4.22) anchored a γ-lactone to the chromone ring system was demonstrated through key HMBC correlations depicted in Figure 4.

**Table 2:** NMR data of diaporthochromones A (**15**), B (**16**) and phomopsis H76 A (**17**).

	15 ^a^		16 ^a^		17 ^b^	
Position	*δ*_H_ (*J* in Hz)	*δ*_C_	*δ*_H_ (int., *J* in Hz)	*δ*_C_	*δ*_H_	*δ*_C_
1		196.9		195.9		200.2
2	3.22 (1H, d, 17.5) 2.86 (1H, d, 17.6)	38.3	3.05 (1H, dd, 17.6, 0.8) 2.90 (1H, brd, 15.1)	37.9	3.11 2.93	39.9
3		83.5		82.9 *		86.6
4a		155.6		158.1		161.5
5		115.2	6.45 * (1H, d, 8.6)	106.9	6.48	109.4
6	7.25 (1H, d, 8.8)	140.2	7.43 * (1H, d, 8.5)	140.8	7.38	142.7
7	6.59 (1H, d, 8.8)	109.9		117.2		118.8
8		161.7		159.0		160.6
8a		107.0		107.2 *		109.6
9	4.22 (1H, d, 3.9)	86.9	4.40 (1H, dd, 1.5,4.4)	86.5 *	4.37	89.3
10	2.67 (1H, m)	29.6	2.84 (1H, m)	29.9	2.79	31.8
11	2.34 (1H, dd, 17.8, 9.0) 2.00 (1H, dd, 18.1, 5.4)	36.2	2.26 (1H, m) 2.23 (1H, m)	36.5	2.75 2.22	38.6
12		176.0		175.6		178.7
14	3.95 (1H, dd, 5.6, 11.6) 3.89 (1H, dd,5.5, 12.3)	62.9	3.94 * (2H, m)	62.4	3.68	65.0
15	1.10 (3H, d, 6.8)	20.5	1.27 (3H, d, 7.0)	20.8	1.15	22.7
1′		197.0		196.7		200.2
2′	3.24 (1H, d, 17.6) 3.10 (1H, d, 17.5)	38.8	3.22 (1H, d, 17.6) 2.96 (1H, d, 17.6, 1.5)	38.2	3.11 2.93	39.9
3′		83.3		82.9 *		86.6
4a′		158.5		158.1		161.5
5′	6.50 (1H, d, 8.3)	107.4	6.46 * (1H, dd, 1.5, 8.5)	106.9	6.48	109.4
6′	7.29 (1H, d, 8.3)	140.1	7.44 * (1H, dd, 1.5, 8.5)	140.8	7.38	142.7
7′		118.6		117.2		118.8
8′		158.4		159.0		160.6
8a′		106.8		107.0 *		109.6
9′	4.33 (1H, d, 3.9)	87.6	4.35 (1H, dd, 1.5, 4.3)	86.4 *	4.37	89.3
10′	2.95 (1H, m)	29.2	2.90 (1H, m)	29.4	2.79	31.8
11′	2.88 (1H, dd, 18.0, 10) 2.22 (1H, dd, 17.6, 4.9)	36.4	2.90 (2H, m)	36.6	2.75 2.22	38.6
12′		175.3		175.8		178.7
14′	3.82 (2H, m)	63.2	3.94 * (1H, m) 3.86 (1H, brd, 11.7)	62.8	3.68	65.0
15′	1.30 (3H, d, 6.8)	20.8	1.30 (3H, d, 6.4)	20.6	1.15	22.7
8-OH	11.73 (1H, s)		12.01 * (1H, s)		11.97	
8′-OH	12.28 (1H, brs)		12.00 * (1H, d, 1.5)		11.97	

^a^ recorded in CDCl_3_ at 500 MHz for ^1^H and at 125 MHz for ^13^C; ^b^ recorded in DMSO-*d*_6_ at 500 MHz for the ^1^H and at 125 MHz for ^13^C [24]; * Interchangeable between monomeric units.

The second monomeric unit associated with compound **15** displayed largely the same HMBC correlations as that described above. The two units could be connected through observation of both H-6 (δ_H_ 7.25) to C-7′ (δ_C_ 118.6) and H-6′ (δ_H_ 7.29) to C-5 (δ_C_ 115.2) HMBC correlations, defining a new dimeric chromone, diaporthochromone A (**15**). Significant chemical shift differences between γ-lactone chiral centers of each monomeric unit suggested an unsymmetrical nature of diaporthochromone A. The 2D ROESY spectrum clearly defined some stereochemical relationships: for example, both monomeric units show strong ROESY correlation between the H-9 methine and the H_3_-15 methyl groups, placing them on the same face (*cis* to one another) of the γ-lactone. That both monomeric units display H-10 to H_2_-14 correlation in the ROESY requires that H-9 and H_3_-15 both be on the opposing face from H_2_-14, reinforcing the H-9/H_3_-15 *cis*-relationship. The major difference between the two monomeric units is the relationship between H-9 and H_2_-2: In the monomeric unit bearing prime distinctions, there is no correlation between the two, while in the other there is, requiring a shift of H-9′ away from the dihydro-γ-pyrone ring system, which can only be achieved by an inverted stereocenter at C-9′ compared to C-9. Thus we propose the relative stereochemistry for diaporthochromone A as 3R^*^,9S^*^,10S^*^,3′S^*^,9′S^*^,10′S^*^.

A second compound was isolated from fungal strain CY-5286 as an isomer of diaporthochromone A (**15**). Diaporthochromone B (**16**) was demonstrated to have the molecular formula C_30_H_30_O_12_ based on HRESIMS (*m/z* at [M + H]^+^ 583.1837; calc’d for C_30_H_31_O_12_ 583.1816). A similar unsymmetrical γ-lactone-substituted chromone dimer was evident from the ^1^H and ^13^C NMR spectra (Table 2). Indeed, except for the two carbons C-5 and C-7, the NMR data is nearly superimposable. Distinguishing **16** are HMBC correlations for overlapping quaternary carbons at δ_C_ 117.2 (C-7/7′) from δ_H_ 12.0 (8/8′-OH), compared to **15** in which two signals are found near 117, quaternary carbons δ_C-7′_ 118.6 and δ_C-5_ 115.2, only one of which (δ_C-7′_ 118.6) correlates to a phenolic proton, 8′-OH in this case, at δ_H_ 12.28. Instead, **15** displays two methine carbons at δ_C-7_ 109.9 and δ_C-5′_ 107.4, in the vicinity of **16**’s overlapping C-5/5′ (δ_C_ 106.9), and one of them, δ_C-7_ 109.0, has HMBC correlation from the phenolic proton 8-OH (δ_H_ 11.73). Thus, where **15** bears a phenolic proton that correlates by HMBC to an aromatic methine, **16** only has phenolic protons correlating to quaternary aromatic carbons. Diaporthochromone B (**16**) therefore bears a linear 7/7′ coupled chromone ring system, analogous to the recently reported phomopsis-H76 A (**17**) [26]. That diaporthochromone B and phomopsis-H76 A are isomeric is evident from variations in the chemical shifts (Table 2), not all of which can be attributed to solvent effects, and their divergent optical rotations: [α]^20^_D_ (CHCl_3_) −24.1 and [α]^25^_D_ (MeOH) +20, respectively. A ROESY spectrum of **16** secured the *cis*-relationship of the 15/15′ methyl groups and their corresponding H-9/9′ methines, analogous to diaporthochromone A (described above) and phomopsis-H76 A. While a small sample size rendered the ROESY from **16** less informative from that of **15**, the H-10 to H-2 correlation, in the absence of an analogous H-10′ to H-2′ correlation, supports an identical stereochemical assignment for the two.

#### 2.5.3. Lipids

**Figure 5:**
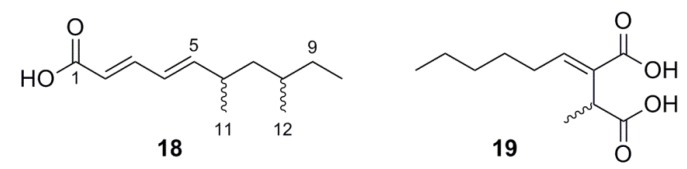
Lipids from Chinese mangrove endophytes.

Two inactive lipids (Figure 5) were found in otherwise active extracts. (2*E*,4*E*)-Dimethyldeca-2,4-dienoic acid (**18**) is a previously unreported compound which was found among active antimalarial cytochalasins from *Xylaria* sp. (strain CY-5331). Similarly, piliformic acid (**19**) [27] was found in strain CY-6884. Neither **18** nor **19** displayed activity against malaria. HRESIMS of **18** indicated a formula of C_12_H_20_O_2_ (*m/z* at [M + H]^+^ 197.1545; calc’d 197.1542), which suggested three unsaturations. The ^13^C NMR chemical shift data indicated carboxylic acid equivalent carbon at δ_C_ 172.5. Sequential olefins followed: H-2 (δ_H_ 5.82) showed correlations to the carboxylate, C-1, and to C-4 (δ_C_ 126.5) in the HMBC spectrum. Correlations observed in the COSY spectrum between H-3 (δ_H_ 7.37) and H-2 and H-4 (δ_H_ 6.20), and H-4 to H-5 (δ_H_ 6.04), secured the C-1 through C-5 dienoate. H-5, in turn, showed HMBC correlation to C-3 (δ_C_ 147.7), C-4 (δ_C_ 126.5), C-6 (δ_C_ 35.0), C-7 (δ_C_ 43.8), and C-11 (δ_C_ 18.9). C-12 (δ_C_ 20.9) is a methyl group substituted on C-8, as suggested by COSY correlation between H-12 (δ_H_ 0.85) and H-8 (δ_H_ 1.31). The terminal methyl protons H-10 (δ_H_ 0.83) correlated to C-8 (δ_H_ 1.31) and C-9 (δ_H_ 1.14) by HMBC. The olefinic stereochemistry of **18** was deduced by analysis of coupling constants (*J*_2,3_ = 15.3 Hz and *J*_4,5_ = 15.2 Hz), supporting the *trans*,*trans*-relationship. The stereochemistry of the methyl substituents is unknown.

## 3. Experimental Section

### 3.1. General Experimental Procedures

Optical rotations were measured on a Rudolph Research Analytical AUTOPOL IV digital polarimeter. IR and UV spectra were measured on Nicolete Avatar 320FT infrared and Hewlett-Packard 8452A diode array instruments, respectively. Medium pressure liquid chromatography was carried out on a Teledyne Isco Combiflash Companion using normal and reverse phase silica gel or C_18_ cartridges, respectively, purchased from Teledyne Isco. High performance liquid chromatography was carried out on semipreparative Phenomenex Luna C_18_(2) reverse phase (250 × 10 mm) and analytical (250 × 4.6 mm) columns using a LC-20A Shimadzu multi-solvent delivery system, a CBM-20A Shimadzu system controller, and a SPD-M20A Shimadzu PDA detector. Low resolution mass spectra were recorded on an Agilent Technologies LC/MSD VL electrospray ionization mass spectrometer. High resolution mass spectra were recorded on an Agilent Technologies LC/MSD TOF electrospray ionization spectrometer. ^1^H and ^13^C NMR spectra were recorded on a Varian Inova instrument operating at 500 MHz for ^1^H, 125 MHz for ^13^C, except 2D ROESY, which were acquired at 600 MHz on a Varian Inova, using residual protonated solvent as ^1^H internal standard or ^13^C absorption lines of solvents for ^13^C internal standard.

### 3.2. Biological Materials

Fungi were isolated from samples collected in mangroves of the South China Sea coast at Mai Po Nature Reserve, or from Hainan Island coastal regions. Segments of approximately 1–2 mm^2^ were plated on malt extract freshwater agar. Fungal growth was examined every day for two weeks and then twice a week for at least one month. A hypha growing out from the incubated plant tissue was cut out and transferred to a fresh malt extract agar plate [15]. Pure axenic endophytic fungal cultures were grown in the commonly used liquid medium which contained 1% w/v glucose, 0.1% w/v yeast extract and 0.2% w/v peptone for 3 weeks for the production of secondary metabolites; samples for crude extract preparation were 30 mL total volume each, while high priority samples were scaled up to 2 L total volume to support fractionation and characterization studies. Cultures were freeze dried and shipped expedited to USF.

### 3.3. Dicerandrol D **(14)**

From 6.4 g of freeze dried mycelia from *Diaporthe* sp. (strain CY-5188), a lipophilic (1:1 CH_2_Cl_2_/CH_3_OH) extract was prepared and fractionated by MPLC and HPLC, using 2% CH_3_OH in CH_2_Cl_2_ on silica gel, to yield 1.6 mg of dicerandrol D (**14**). Yellow amorphous solid. [α]^20^_D_ +12.6 (*c* 0.02 CHCl_3_); ^1^H NMR (500 MHz, CDCl_3_) δ_H_ 14.23 (enol, s, 1H), 13.98 (enol, s, 1H), 11.69 (phenol, s, 1H), 11.66 (phenol, s, 1H), 7.41 (H-3, d, *J* = 8.9 Hz, 1H), 7.31 (H-3′, *J* = 8.8 Hz, 1H), 6.57 (H-4, d, *J* = 8.9 Hz, 1H), 6.52 (H-4′, d, *J* = 8.8 Hz, 1H), 5.83 (H-5, s, 1H), 5.61 (H-5′, s, 1H), 5.03 (H-12, d, *J* = 12.6 Hz, 1H), 4.01 (H-12′, dd, *J* = 13.6, 5.1 Hz, 1H), 3.62 (H-12, d, *J* = 12.8 Hz, 1H), 3.39 (H-12′, dd, *J* = 13.5, 10.0 Hz, 1H), 2.56-2.29 (H-6/6′, H-7/7′, m, 6H), 2.21 (H-14, s, 3H), 2.18 (H-14′, d, 3H), 1.63 (H-16′, s, 3H), 1.07 (H-11, d, *J* = 6.2 Hz, 3H), 1.04 (H-11′, d, *J* = 5.7 Hz, 3H); ^13^C NMR (125 MHz, CDCl_3_) δ_C_ 187.9, 187.5 (C-9/9′), 177.6, 177.1 (C-8/8′), 170.8, 169.9 (C-13/13′), 169.8 (C-15′), 161.5, 161.3 (C-1/1′), 154.1, 154.1 (C-4a/4a′), 141.6, 141.1 (C-3/3′), 115.1, 115.0 (C-2/2′), 109.8, 108.8 (C-4/4′), 106.2, 106.0 (C-9/9′), 101.0, 99.7 (C-8a/8a′), 82.2, 80.9 (C-10a/10a′), 70.0, 70.0 (C-5/5′), 65.2, 65.4 (C-12/12′), 33.4, 33.4 (C-7/7′), 28.1, 27.7 (C-6/6′), 21.1, 21.0 (C-14/14′), 17.7, 17.6 (C-11/11′), 20.1 (C-16′); HRESIMS *m*/*z*: [M + H]^+^ 709.2137, [M + Na]^+^ 731.1967 (calc’d for C_36_H_37_O_15_ 709.2132, C_36_H_36_NaO_15_, 731.1952).

### 3.4. Diaporthochromones (**15** and **16**)

From 4.9 g of freeze dried mycelia from *Diaporthe* sp. (strain CY-5286), 2.1 g of lipophilic extract (1:1 CH_2_Cl_2_/CH_3_OH) was fractionated first on normal phase MPLC using a CH_2_Cl_2_ gradient with increasing CH_3_OH, then repeated HPLC (Figure S8) to achieve separation of **15** and **16**, finally yielding 1.2 and 1.0 mg, respectively.

Diaporthochromone A (**15**): White amorphous solid. [α]^20^_D_ −84.6 (*c* 0.1 CHCl_3_); CD (*c* 0.01, MeOH) λ_max_ nm (ε) 245 (−30.5), 286 (−22.7); ^1^H NMR and ^13^C NMR (Table 2); HRESIMS *m*/*z*: [M + H]^+^ 583.1842, [M + Na]^+^ 605.1630 (calc’d for C_30_H_31_O_12_ 583.1816; C_30_H_30_NaO_12_ 605.1635).

Diaporthochromone B (**16**): Yellow amorphous solid. [α]^20^_D_ −24.1 (*c* 0.1 CHCl_3_); CD (*c* 0.01, MeOH) λ_max_ nm (ε) 250 (+14.1), 293 (4.2); ^1^H NMR and ^13^C NMR (Table 2); HRESIMS *m*/*z*: [M + H]^+^ 583.1837 (calc’d for C_30_H_31_O_12_ 583.1816).

### 3.5. (2*E*,4*E*)-6,8-Dimethyldeca-2,4-dienoic Acid (**18**)

From 4.89 g of freeze dried CY-5331 mycelia, 2.06 g of lipophilic (1:1 CH_2_Cl_2_/CH_3_OH) extract were obtained. Silica gel MPLC using 50% EtOAc/hexane yielded 12 fractions, including a 226 mg fraction 2. Fraction 2, after further MPLC (30% EtOAc/hexane) and HPLC using 20% EtOAc/hexane, yielded 7 mg of **18**. Yellow oil. [α]^20^_D_ −20.1 (*c* 0.1 CHCl_3_); ^1^H NMR (500 MHz, CDCl_3_) δ_H_ 7.37 (H-3, dd, *J* = 15.3, 10.9 Hz, 1H), 6.20 (H-4, dd, *J* = 15.2, 10.9 Hz, 1H), 6.04 (H-5, dd, *J* = 15.2, 8.3 Hz, 1H), 5.82 (H-2, d, *J* = 15.4 Hz, 1H), 2.38 (H-6, m, 1H), 1.31 (H_2_-7, H-8, m, 3H), 1.14 (H-9, m, 2H), 1.03 (H-11, d, *J* = 6.6 Hz, 3H), 0.85 (H-12, d, *J* =7.3 Hz, 3H), 0.83 (H_3_-10, m, 3H); ^13^C NMR (125Hz, CDCl_3_) δ_C_ 172.5 (C-1), 152.0 (C-5), 147.7 (C-3), 126.5 (C-4), 118.2 (C-2), 43.8 (H-7), 35.0 (C-6), 32.0 (C-8), 29.9 (C-9), 20.9 (C-12), 18.9 (C-11), 11.2 (C-10); HRESIMS *m*/*z*: [M + H]^+^ 197.1545, [M + Na]^+^ 219.1365 (calc’d for C_12_H_21_O_2_, 197.1542; C_12_H_20_NaO_2_, 219.1361).

### 3.6. Malaria Assay

Malaria screening was conducted as previously reported [13].

### 3.7. *In Vitro* Toxicity Assay

Cell line A-549 (adenocarcinomic human alveolar epithelial cells) was cultured in F-12K Nutrient Mixture (Kaighn’s Modification) media containing L-glutamine, supplemented with 10% fetal bovine serum and 1% penicillin-streptomycin. For the assay, A549 cells were diluted to 1.33 × 105 cells/mL in DMEM F12 media with l-glutamine, without HEPES or phenol red, and supplemented with 2% fetal bovine serum and 1% penicillin-streptomycin. Test compounds at 2 mg/mL in DMSO were diluted 1:200 then serially diluted in duplicate over 11 concentrations. In 96-well plates, a volume of 90 µL/well of A549 cells was added on top of 25 µL/well of the test compound. Final concentration of A549 cells was 12,000 cells per well and the final starting concentration for test compounds was 10 µg/mL. A Beckman-Coulter Biomek 3000 was used to dispense cells and prepare and dispense test compounds to the 96 well plates. Positive and negative controls were included on each assay plate. Plates were incubated for 72 h at 37 °C and 5% CO_2_. After the incubation period, cell proliferation was assessed using Promega’s CellTiter 96 Aqueous One Solution Cell Proliferation Assay reagent. Into each well 20 µL of reagent was added followed by incubation for 3.5 h at 37 °C and 5% CO_2_. A Molecular Devices Spectramax M2e plate reader was used to read absorbance at 490 nM. IC_50_ values were determined using a custom database manager (Dartaspects Corporation, Glencoe, CA, USA) by the use of nonlinear regression analysis.

## 4. Conclusions

A significant number of endophytic fungal extracts have been evaluated for antimalarial activity. The stringent bioassay restrictions used to advance extracts and fractions, using both malaria and cytotoxicity data, limited resource intensive scale-up and fractionation studies to roughly 1% of fungi studied. Nonetheless, the subset of fungal extracts described in this paper, derived from Hong Kong and Taiwan mangroves, yielded several new compounds, one of which, dicerandrol D (**14**), met our target criteria of nanomolar malaria activity with at least 10-fold less cytotoxicity.

## Supplementary Files

Supplementary File 1 Supplementary Information (PDF, 1713 KB)

## References

[1] Rateb M.E., Ebel R. (2011). Secondary metabolites of fungi from marine habitats. Nat. Prod. Rep..

[2] Xu J. (2011). Biomolecules produced by mangrove-associated microbes. Curr. Med. Chem..

[3] Wu J., Xiao Q., Xu J., Li M.Y., Pan J.Y., Yang M.H. (2008). Natural products from true mangrove flora: Source, chemistry and bioactivities. Nat. Prod. Rep..

[4] Omar S., Godard K., Ingham A., Hussain H., Wongpanich V., Pezzuto J., Durst T., Eklu C., Gbeassor M., Sanchez-Vindas P. (2003). Antimalarial activities of gedunin and 7-methoxygedunin and synergistic activity with dillapiol. Ann. Appl. Biol..

[5] Castillo U., Harper J.K., Strobel G.A., Sears J., Alesi K., Ford E., Lin J., Hunter M., Maranta M., Ge H. (2003). Kakadumycins, novel antibiotics from *Streptomyces* sp. NRRL, 30566, an endophyte of *Grevillea pteridifolia*. FEMS Microbiol. Lett..

[6] Isaka M., Suyarnsestakorn C., Tanticharoen M., Kongsaeree P., Thebtaranonth Y. (2002). Aigialomycins A–E, new resorcylic macrolides from the marine mangrove fungus *Aigialus parvus*. J. Org. Chem..

[7] Feller I.C., Lovelock C.E., Berger U., McKee K.L., Joye S.B., Ball M.C. (2010). Biocomplexity in mangrove ecosystems. Annu. Rev. Mar. Sci..

[8] Mangrove Forest Threats. http://wwf.panda.org/about_our_earth/blue_planet/coasts/mangroves/mangrove_threats/.

[9] Alongi D.M. (2002). Present state of future and the world’s mangrove forests. Environ. Conserv. J..

[10] Valiela I., Bowen J.L., York J.K. (2001). Mangrove forests: one of the world’s threatened major tropical environments. BioScience.

[11] Polidoro B.A., Carpenter K.E., Collins L., Duke N.C., Ellison A.M., Farnsworth E.J., Fernando E.S., Kathiresan K., Koedam N.E., Livingstone S.R. (2010). The loss of species: Mangrove extinction risk and geographic areas of global concern. PLoS One.

[12] Mace G.M., Norris K., Fitter A.H. (2011). Biodiversity and ecosystem services: A multilayered relationship. Trends Ecol. Evol..

[13] Lebar M.D., Hahn K.N., Mutka T., Maignan P., van Olphen A., Kyle D.E., McClintock J.B., Amsler C.D., Baker B.J. (2011). CNS and antimalarial activity of synthetic meridianin and psammopemmin analogs. Bioorg. Med. Chem..

[14] Kowalski T., Kehr R.D. (1992). Endophytic fungal colonization of branch bases in several forest tree species. Sydowia.

[15] Pang K.L., Vrijmoed L.L.P., Goh T.K., Plaingam N., Jones G.E.B. (2008). Fungal endophytes associated with *Kandelia candel* (Rhizophoraceae) in Mai Po Nature Reserve, Hong Kong. Bot. Mar..

[16] Edwards R.L., Maitland J., Whalley A.J.S. (1989). Metabolites of the higher fungi. Part 24. Cytochalasin N, O, P, Q, and R. New cytochalasins from the fungus *Hypoxylon terricola* Mill. J. Chem. Soc. Perkin Trans. I.

[17] Liu J., Jianwen T., Dong Z., Ding Z., Wang X., Liu P. (2002). Neoengleromycin, a novel compound from *Engleromyces goetzii*. Helv. Chem. Acta.

[18] Espada A., Rivera-Sagredo A., de la Fuente J.M., Hueso-Rodriguez J.A., Elson S.W. (1997). New cytochalasins from the fungus *Xylaria hypoxylon*. Tetrahedron.

[19] Konig G.M., Wright A.D., Angerhofer C.K. (1996). Antimalarial diterpene isonitriles, isothiocyanates and isocyanates from the tropical marine sponge *Cymbastela hooperi*. J. Org. Chem..

[20] Namikoshi M., Akano K., Meguro S., Kasuga I., Mine Y., Takahashi T., Kobayashi H. (2001). A new macrocyclic trichothecene, 12,13-deoxyroridin E, produced by the marine-derived fungus *Myrothecium roridum* collected in Palau. J. Nat. Prod..

[21] Pontius A., Mohamed I., Krick A., Kehraus S., Konig G.M. (2008). Aromatic polyketides from marine algicolous fungi. J. Nat. Prod..

[22] Kokubun T., Bridge P.D., Simmonds M.S.J. (2003). Dihydroisocoumarins and a tetralone from *Cytospora eucalypticola*. Phytochemistry.

[23] Kamisuki S., Ishimaru C., Onoda K., Kuriyama I., Ida N., Sugawara F., Yoshida H., Mizushina Y. (2007). Nodulisporol and nodulisporone, novel specific inhibitors of human DNA polymerase lambda from a fungus, *Nodulisporium* sp.. Bioorg. Med. Chem..

[24] Ma W.S. (2011). Natural Product Drug Discovery against Tropical Diseases. Ph.D. Thesis.

[25] Wagenaar M.M., Clardy J. (2001). Dicerandrols, new antibiotic and cytotoxic dimers produced by the fungus *Phomopsis longicolla* isolated from an endangered mint. J. Nat. Prod..

[26] Yang J.F., Xu F., Huang C.H., Li J., She Z.G., Pei Z., Lin Y.C. (2010). Metabolites from the mangrove endophytic fungus *Phomopsis* sp. (#zsu-H76). Eur. J. Org. Chem..

[27] Anderson J.R., Edward R.L., Whalley A.C.J. (1985). Metabolites of the higher fungi. Part 22. 2-Butyl-3-methylsuccinic acid and 2-hexylidene-3-methylsuccinic acid from xylariaceous fungi. J. Chem. Soc. Perkin Trans. I.

