# Supplementary material for: Screening Mangrove Endophytic Fungi for Antimalarial Natural Products
Source: Mar Drugs. 2013 Dec 12;11(12):5036–50. doi: 10.3390/md11125036 (PMC3877901; doi:10.3390/md11125036)

Supplementary Information

Figure S1. Isolation scheme for **13** and **14**.
Figure S2. ^{1}H NMR spectrum of **14** (500 MHz, $CDCl_3$).
Figure S3. ^{13}C NMR spectrum of **14** (125 MHz, $CDCl_3$).
Figure S4. COSY spectrum of **14** (500 MHz, $CDCl_3$).
Figure S5. HSQC spectrum of **14** (500 MHz, $CDCl_3$).
Figure S6. HMBC spectrum of **14** (500 MHz, $CDCl_3$).
Figure S7. ROESY spectrum of **14** (500 MHz, $CDCl_3$).
Figure S8. Isolation scheme for **15** and **16**.
Figure S9. ^{1}H NMR spectrum of **15** (500 MHz, $CDCl_3$).
Figure S10. ^{13}C NMR spectrum of **15** (125 MHz, $CDCl_3$).
Figure S11. COSY spectrum of **15** (500 MHz, $CDCl_3$).
Figure S12. HSQC spectrum of **15** (500 MHz, $CDCl_3$).
Figure S13. HMBC spectrum of **15** (500 MHz, $CDCl_3$).
Figure S14. Expansion of ROESY spectrum of **15**, 0–5 ppm (600 MHz, $CDCl_3$).
Figure S15. ^{1}H NMR spectrum of **16** (500 MHz, $CDCl_3$).
Figure S16. ^{13}C NMR spectrum of **16** (125 MHz, $CDCl_3$).
Figure S17. COSY spectrum of **16** (500 MHz, $CDCl_3$).
Figure S18. HSQC spectrum of **16** (500 MHz, $CDCl_3$).
Figure S19. HMBC spectrum of **16** (500 MHz, $CDCl_3$).
Figure S20. Expansion of ROESY spectrum of **16**, 1.0–5.0 ppm (600 MHz, $CDCl_3$).
Figure S21. Isolation scheme for **18**.
Figure S22. ^{1}H NMR spectrum of **18** (500 MHz, $CDCl_3$).
Figure S23. ^{13}C and DEPT NMR spectra of **18** (125 MHz, $CDCl_3$).
Figure S24. COSY spectrum of **18** (500 MHz, $CDCl_3$).
Figure S25. HSQC spectrum of **18** (500 MHz, $CDCl_3$).
Figure S26. HMBC spectrum of **18** (500 MHz, $CDCl_3$).

Figure S1. Isolation scheme for **13** and **14**. Red highlights indicate antimalarial activity.

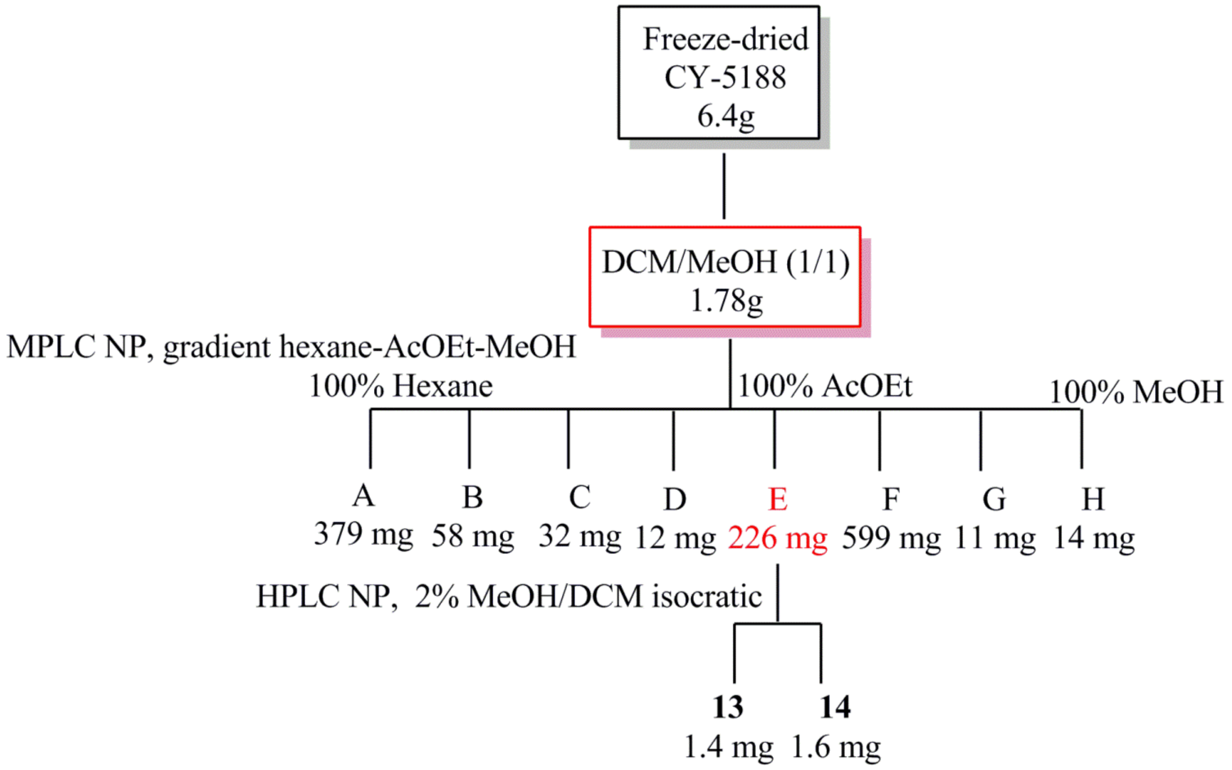

Figure S2. ^{1}H NMR spectrum of **14** (500 MHz, $CDCl_3$).

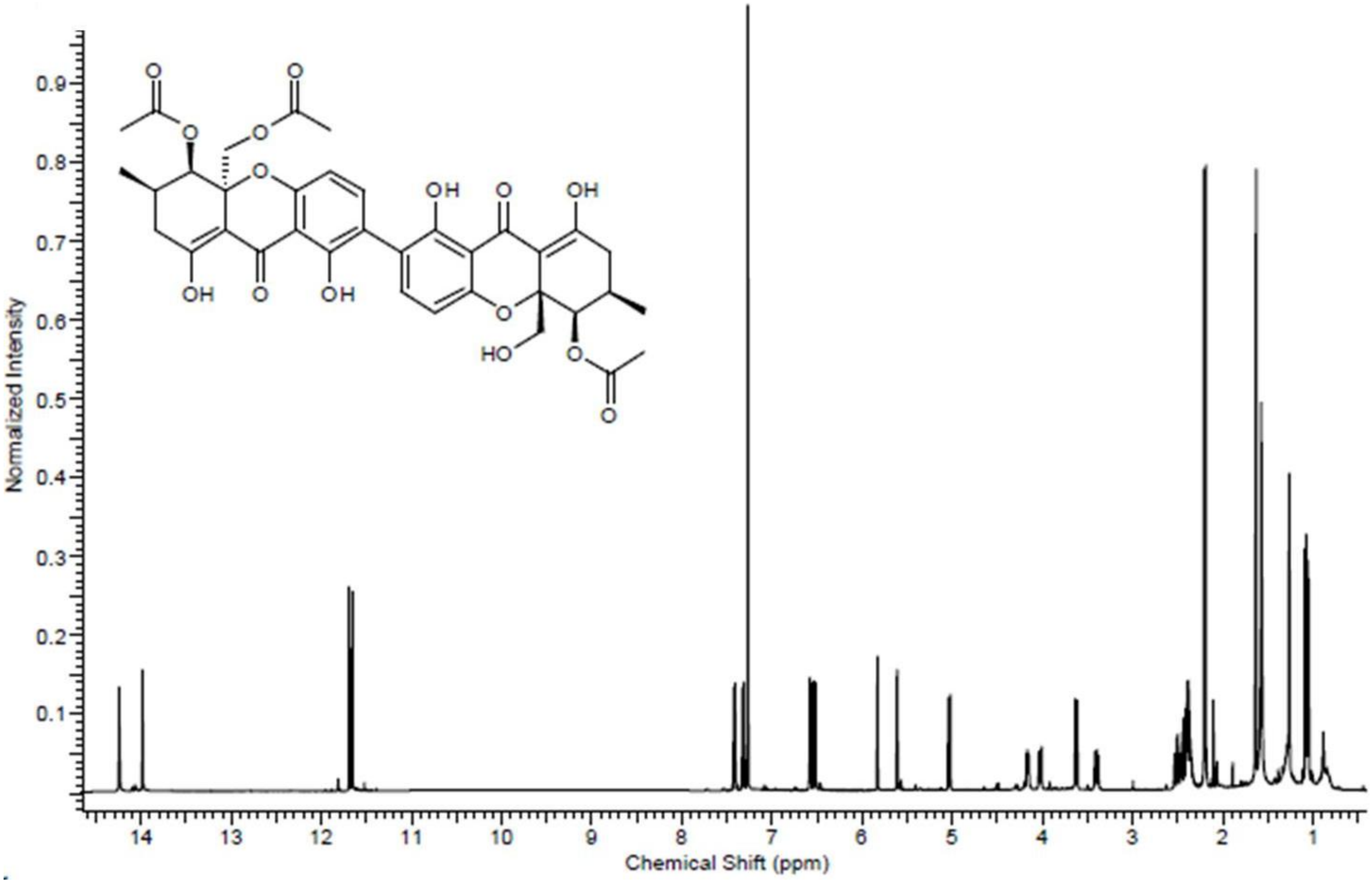

Figure S3. ^{13}C NMR spectrum of **14** (125 MHz, $CDCl_3$).

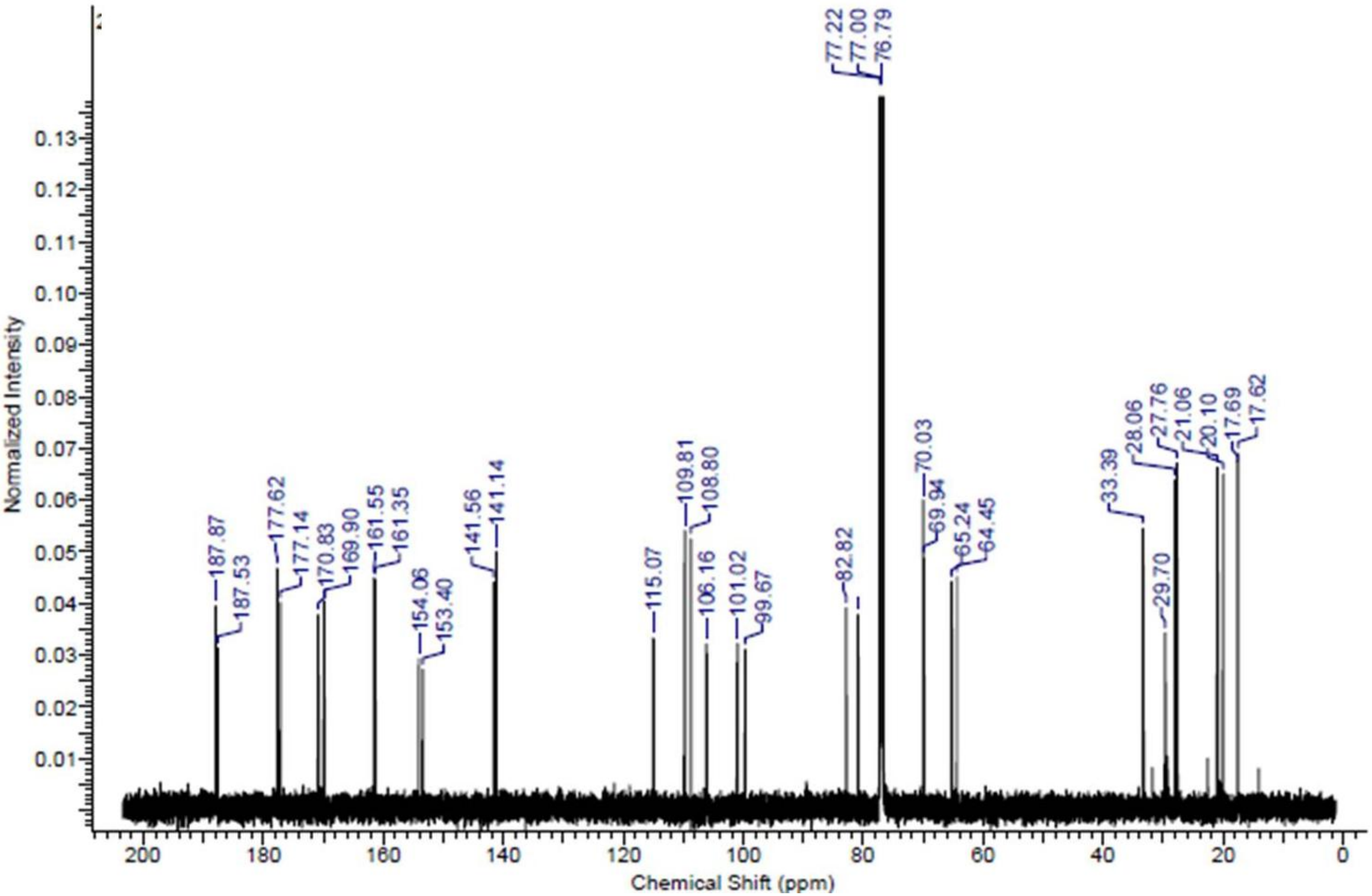

Figure S4. COSY spectrum of **14** (500 MHz, $CDCl_3$).

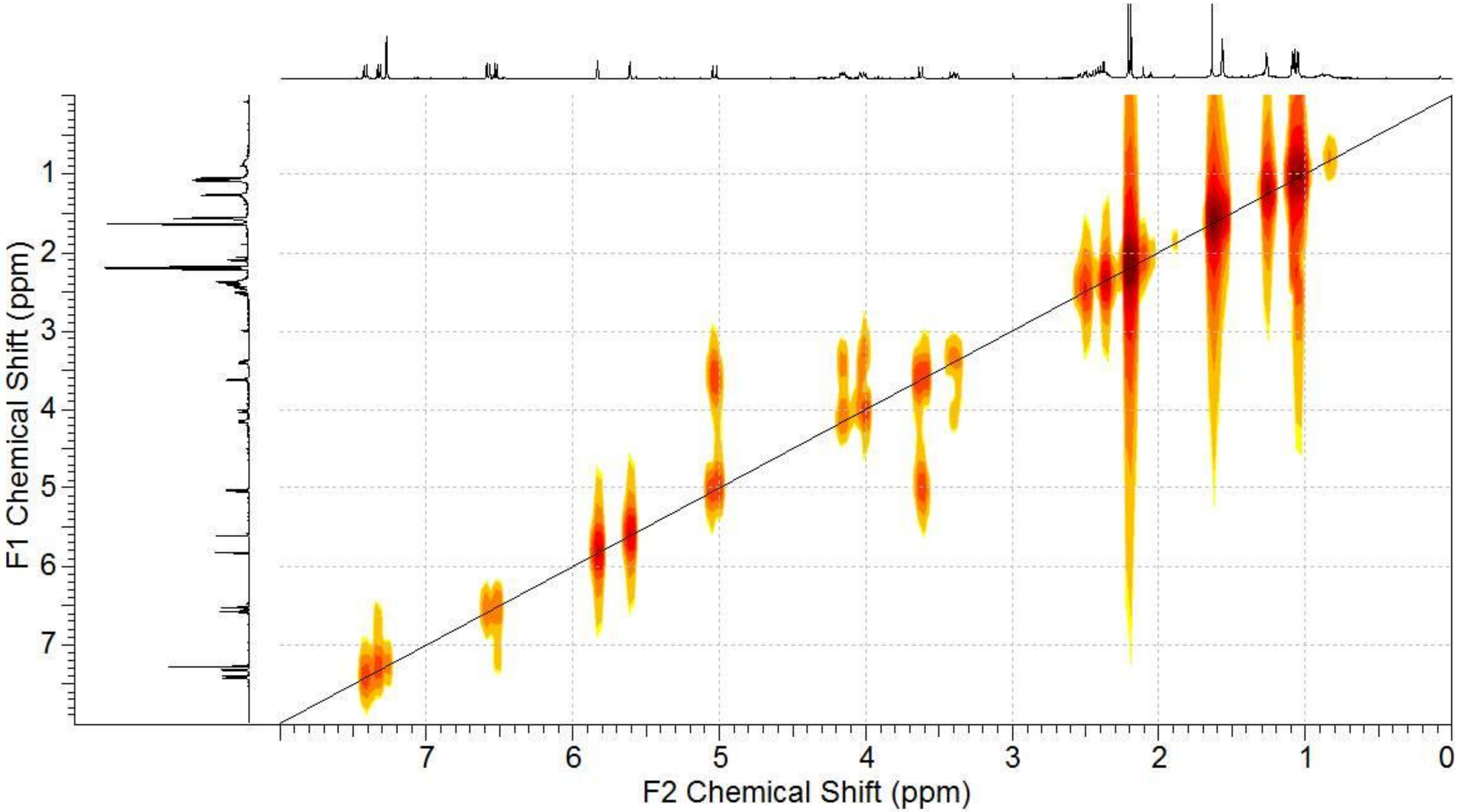

Figure S5. HSQC spectrum of **14** (500 MHz, $CDCl_3$).

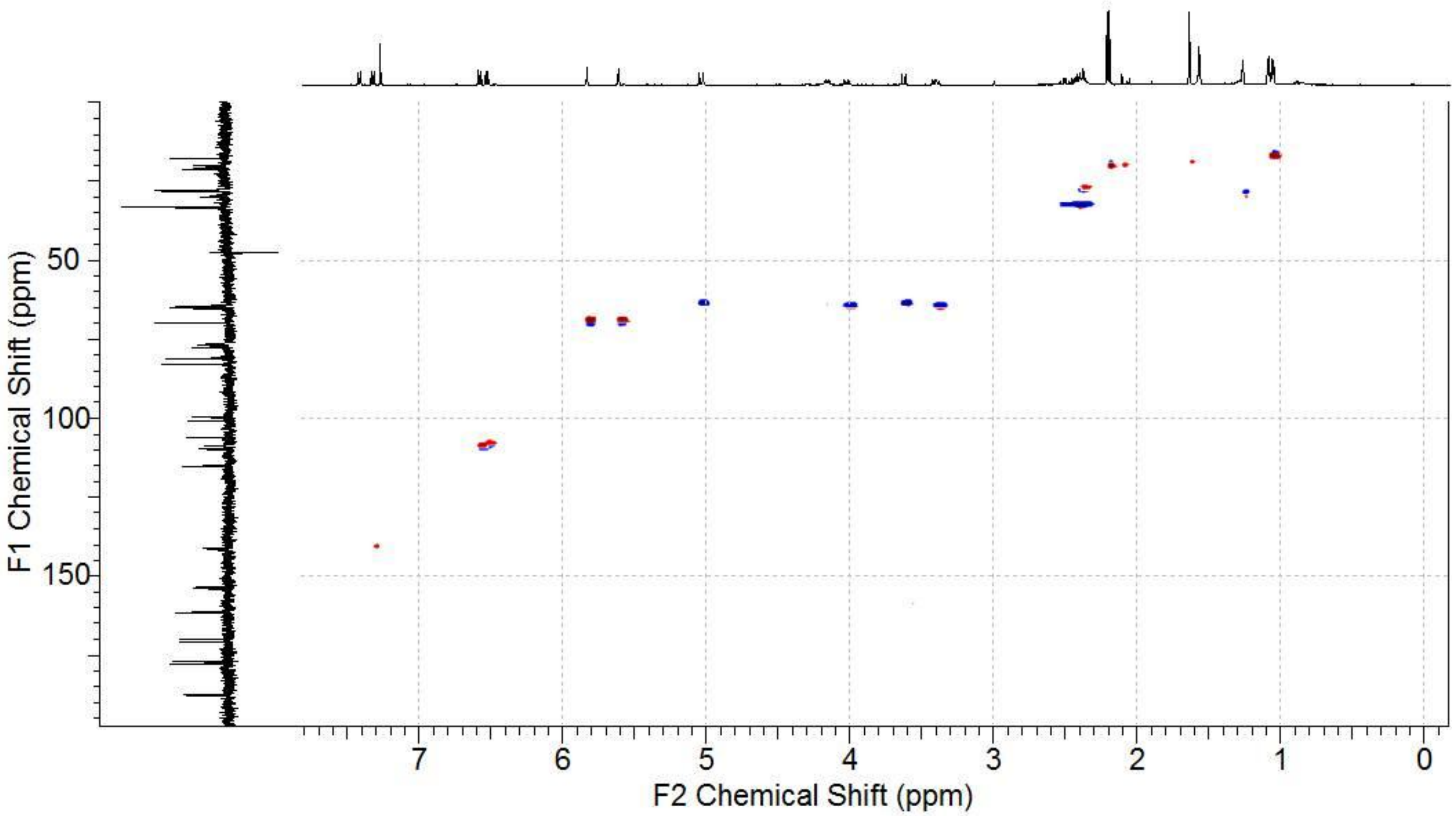

Figure S6. HMBC spectrum of **14** (500 MHz, $CDCl_3$).

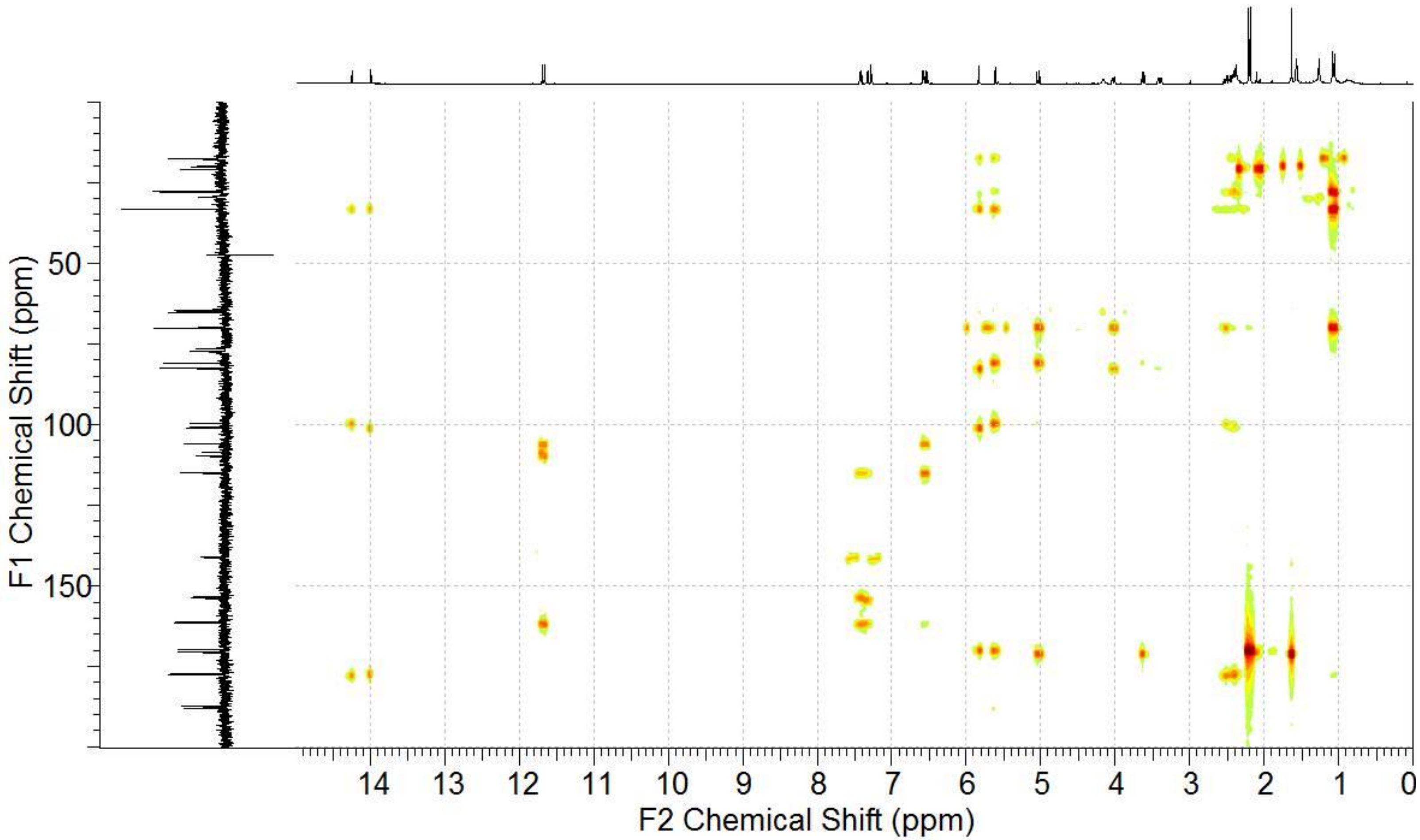

Figure S7. ROESY spectrum of **14** (500 MHz, $CDCl_3$).

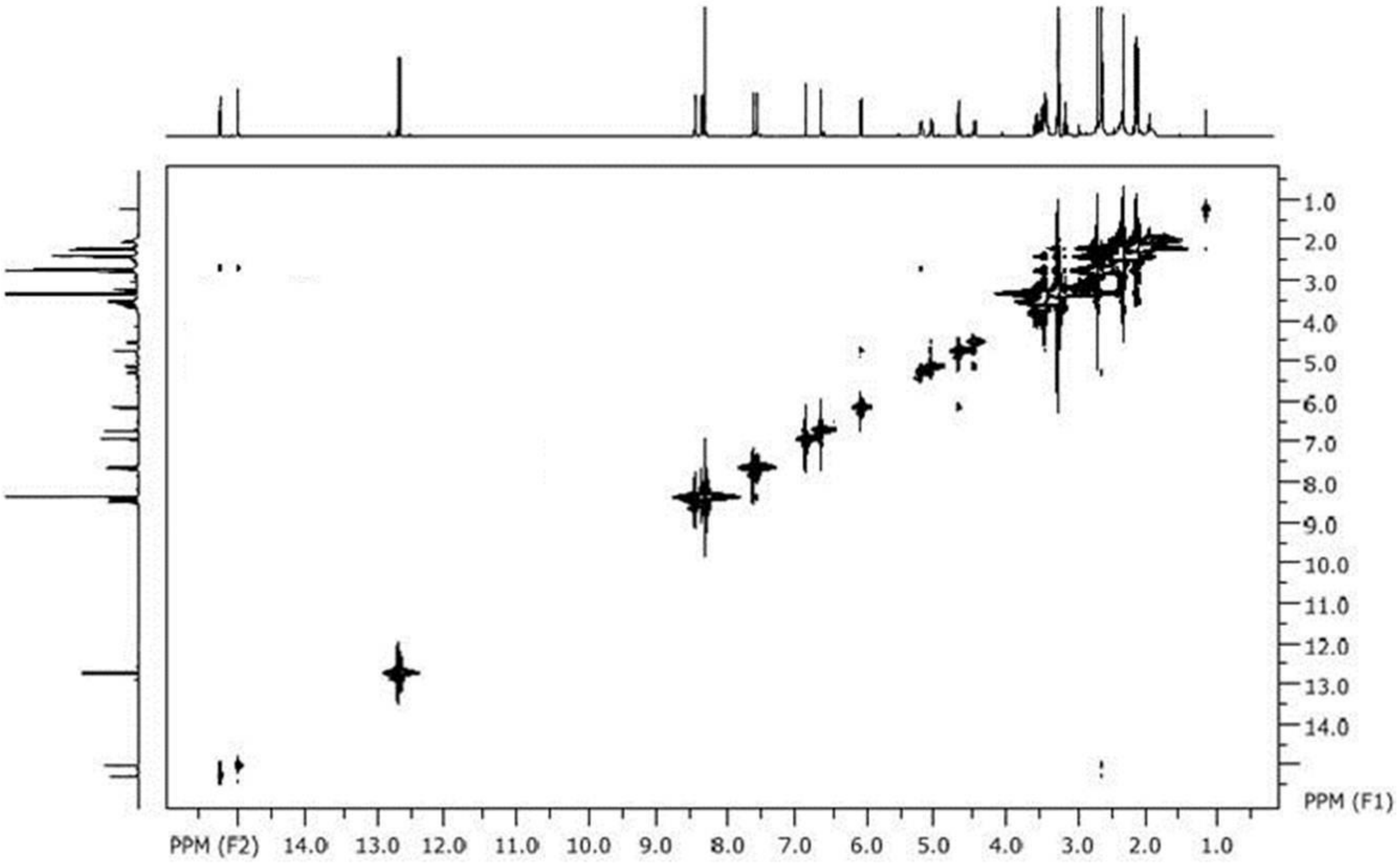

Figure S8. Isolation scheme for **15** and **16**. Red highlights indicate antimalarial activity.

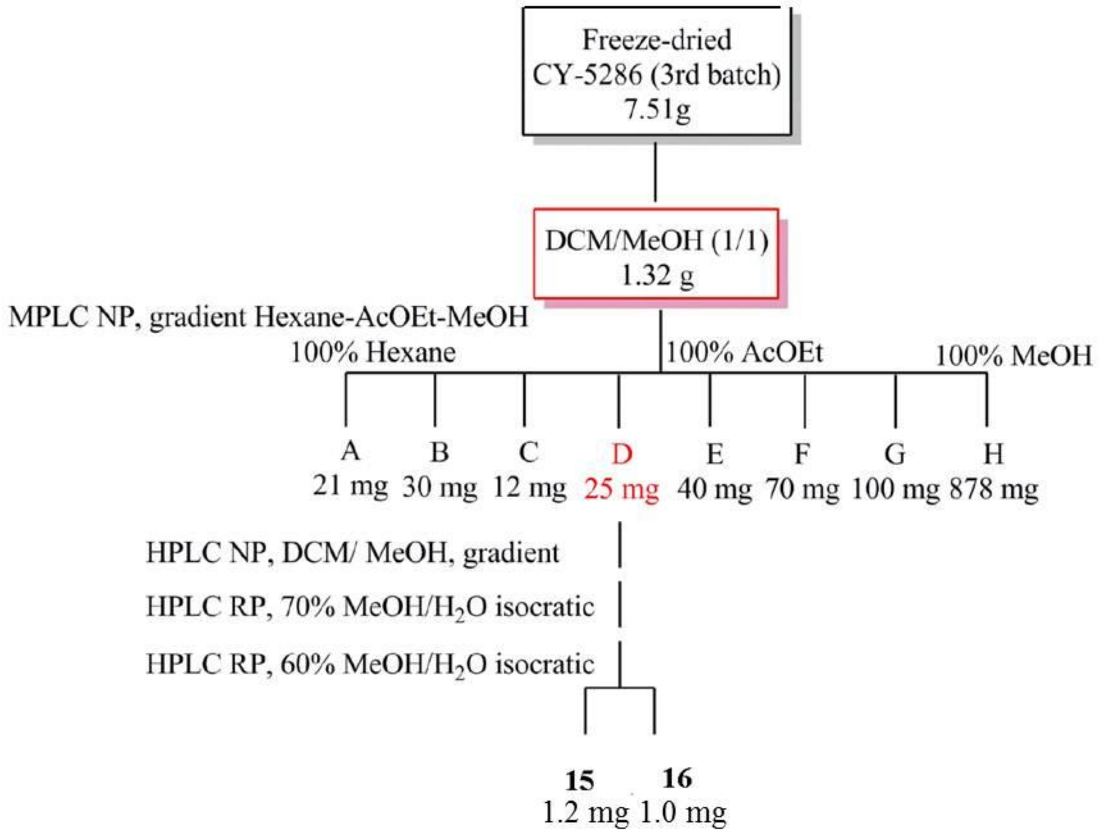

Figure S9. ^{1}H NMR spectrum of **15** (500 MHz, $CDCl_3$).

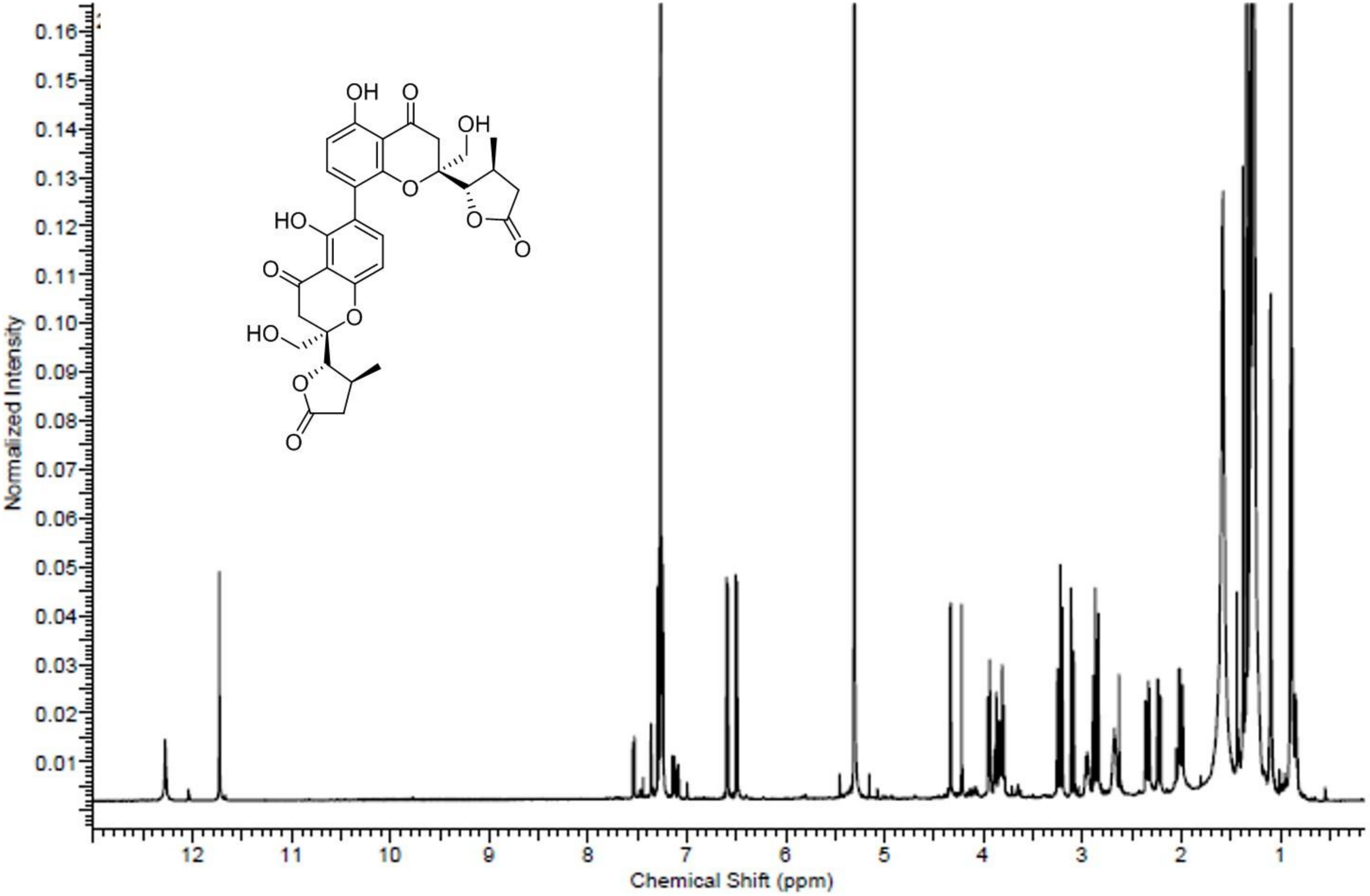

Figure S10. ^{13}C NMR spectrum of **15** (125 MHz, $CDCl_3$).

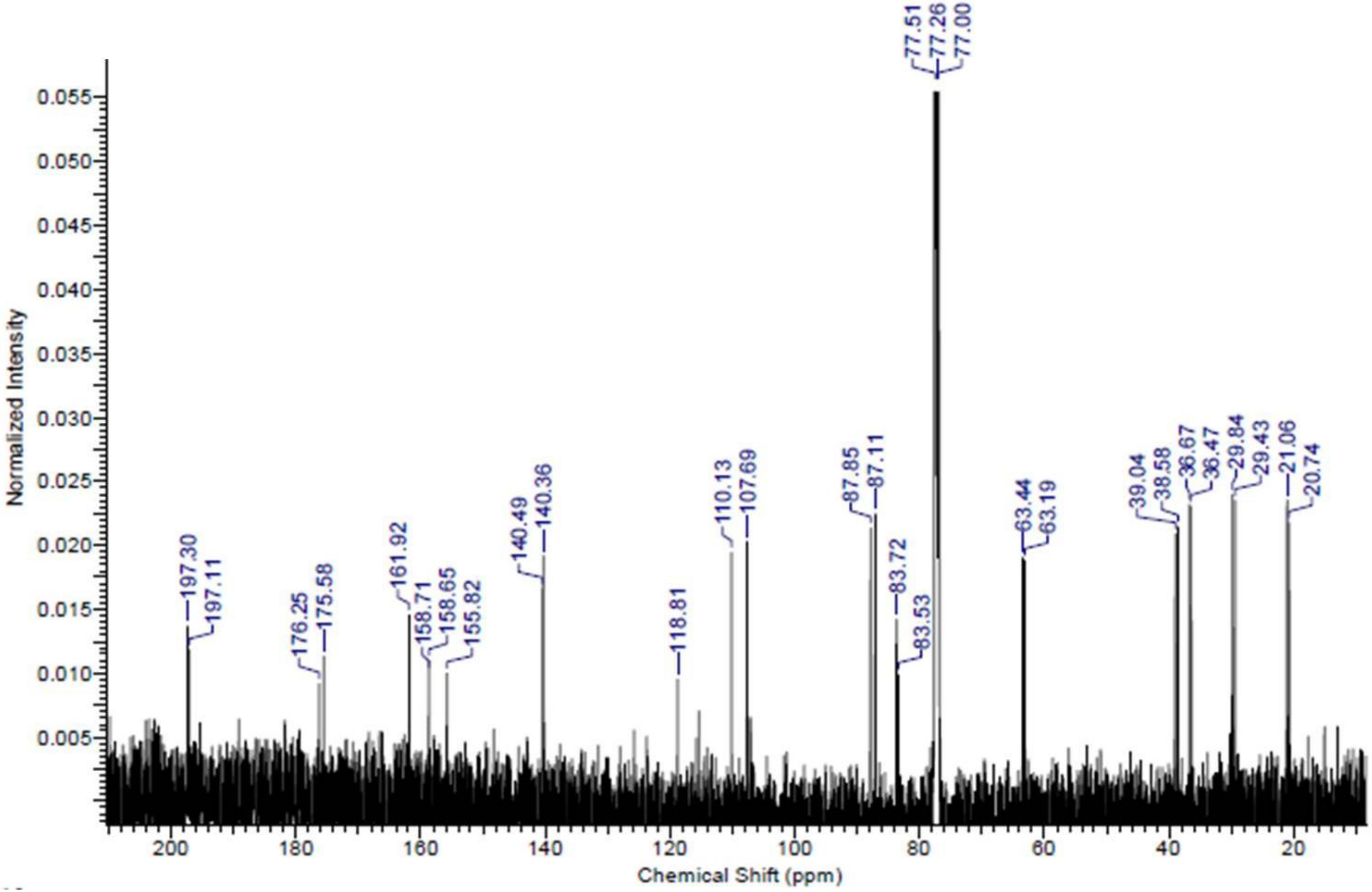

Figure S11. COSY spectrum of **15** (500 MHz, $CDCl_3$).

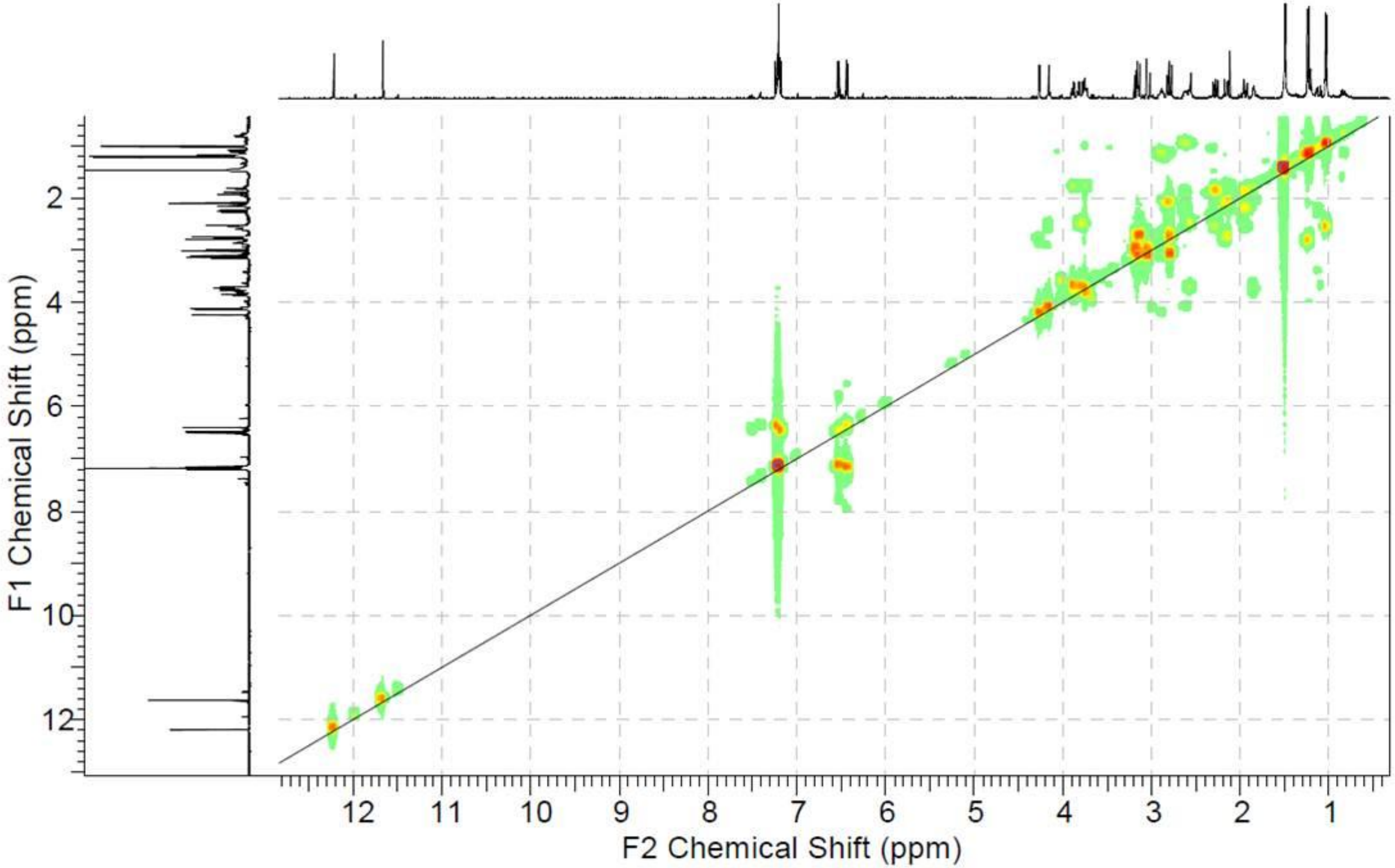

Figure S12. HSQC spectrum of **15** (500 MHz, $CDCl_3$).

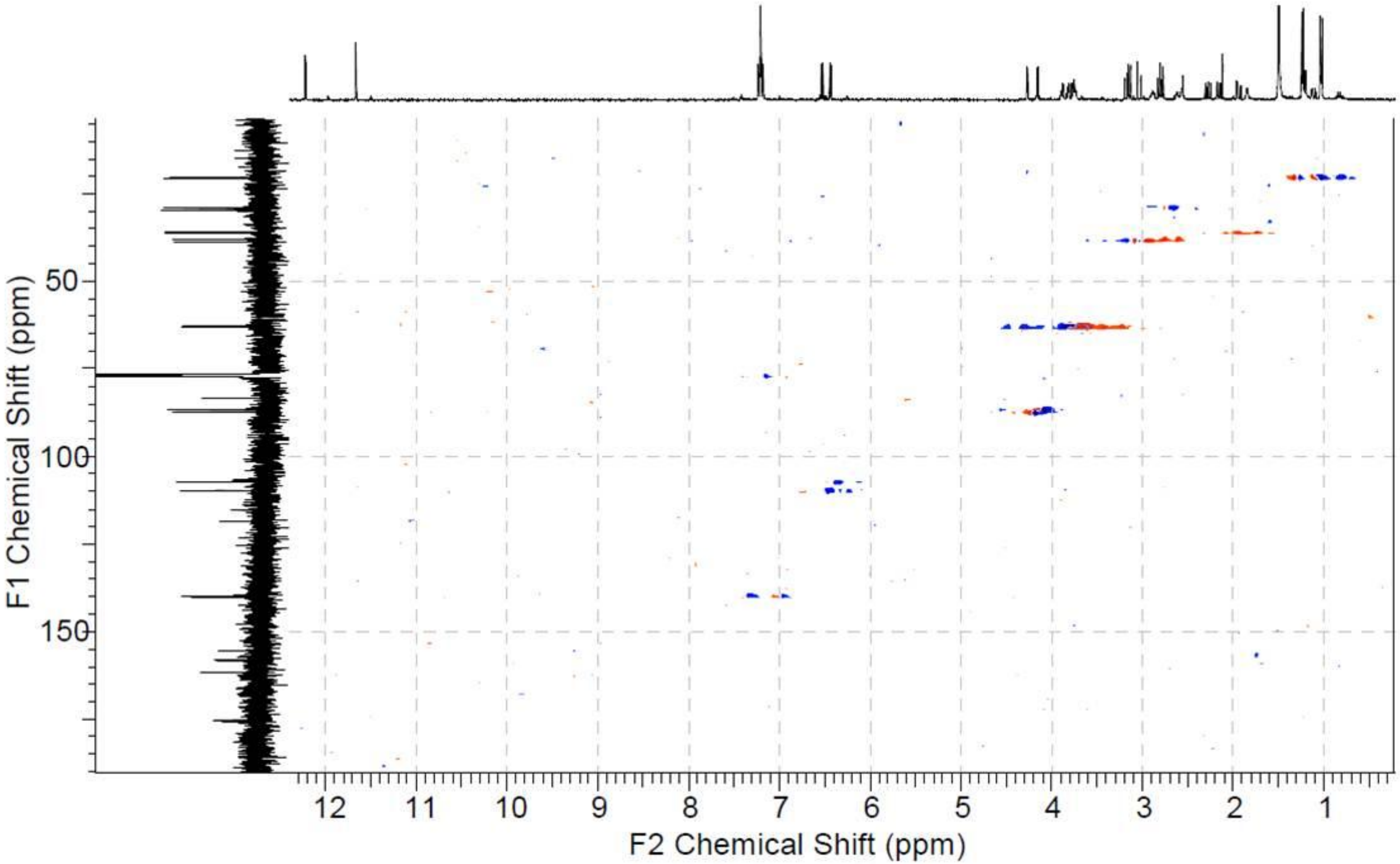

Figure S13. HMBC spectrum of **15** (500 MHz, $CDCl_3$).

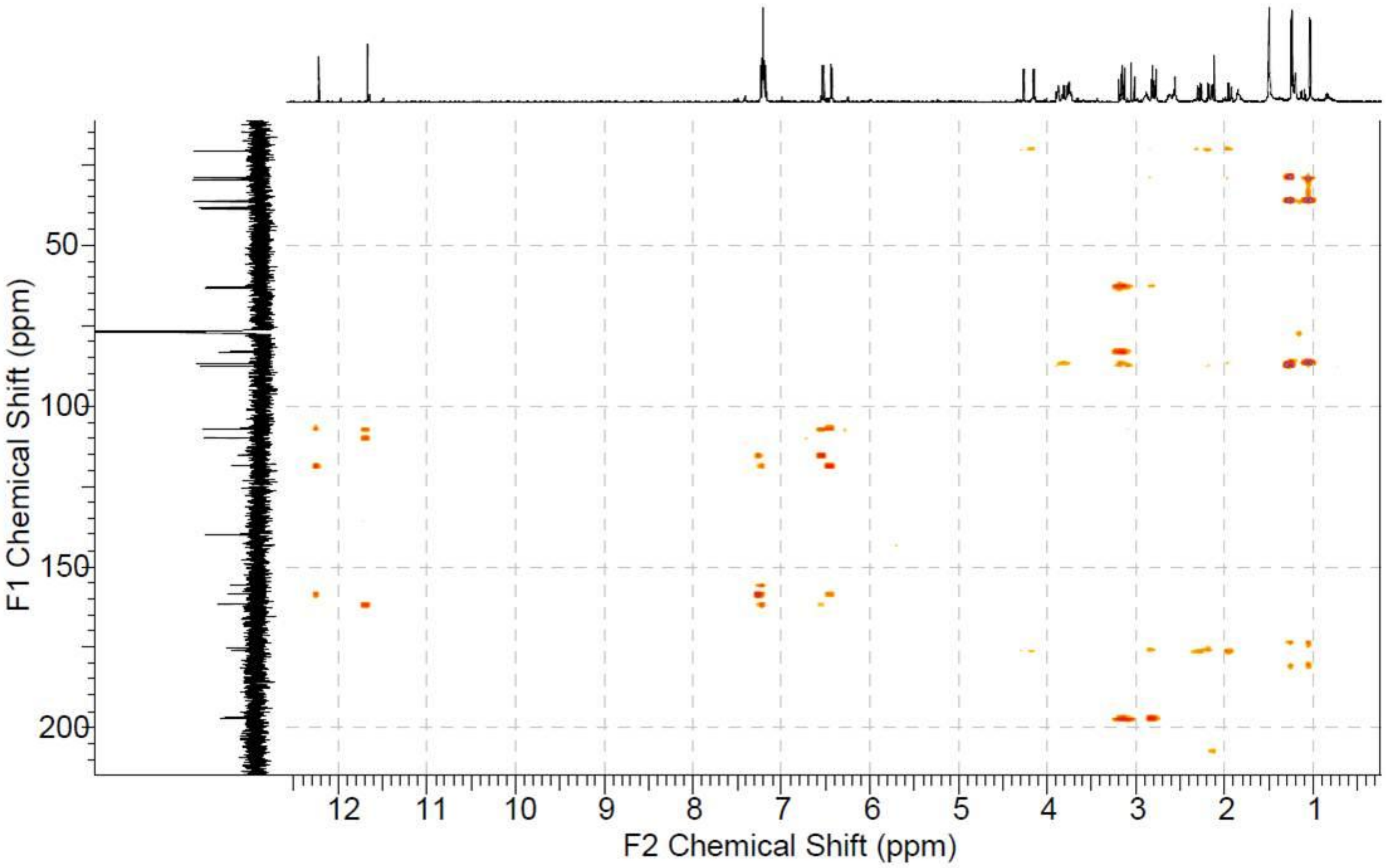

Figure S14. Expansion of ROESY spectrum of **15**, 0–5 ppm (600 MHz, $CDCl_3$).

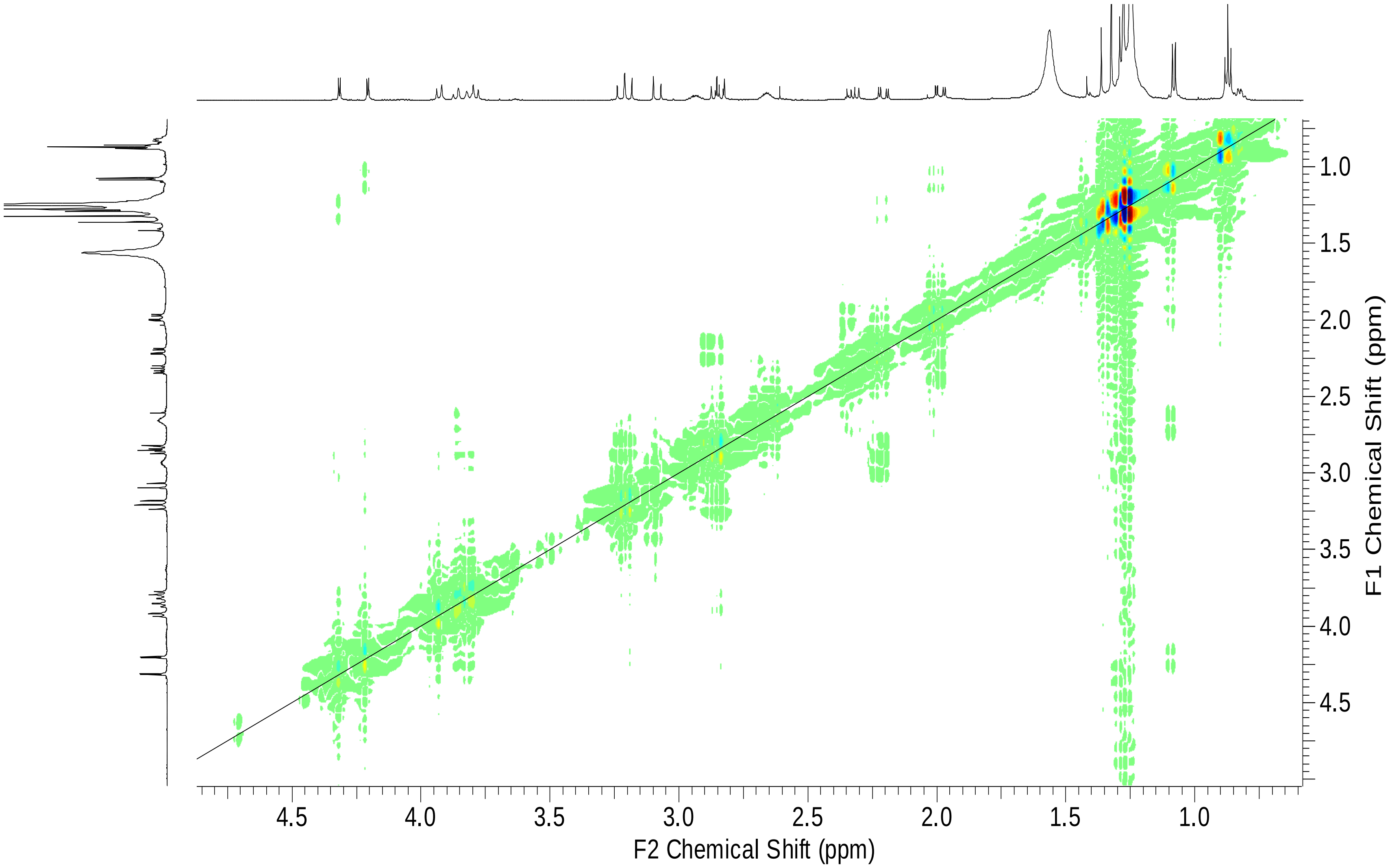

Figure S15. ^{1}H NMR spectrum of **16** (500 MHz, $CDCl_3$).

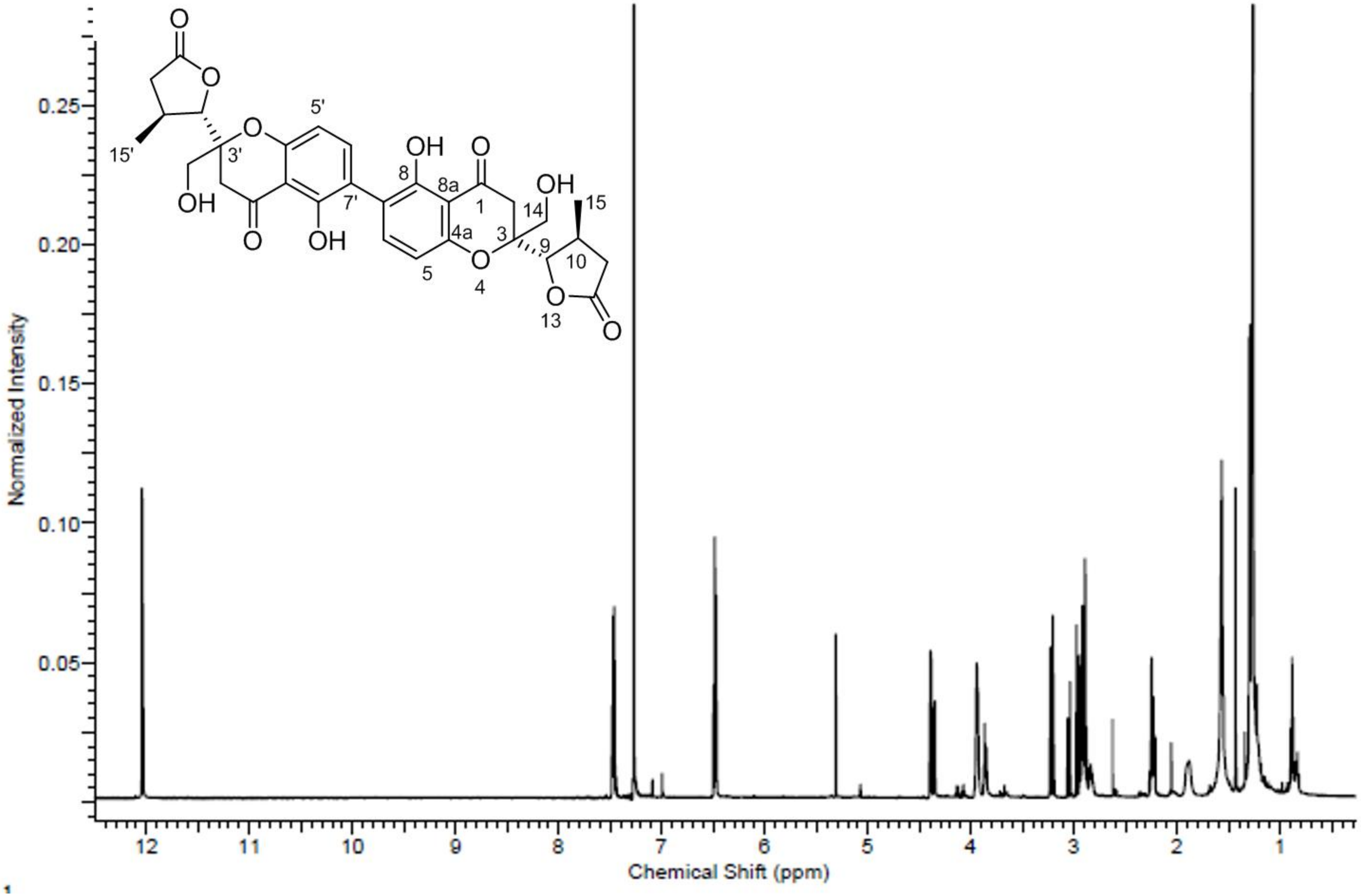

Figure S16. ^{13}C NMR spectrum of **16** (125 MHz, $CDCl_3$).

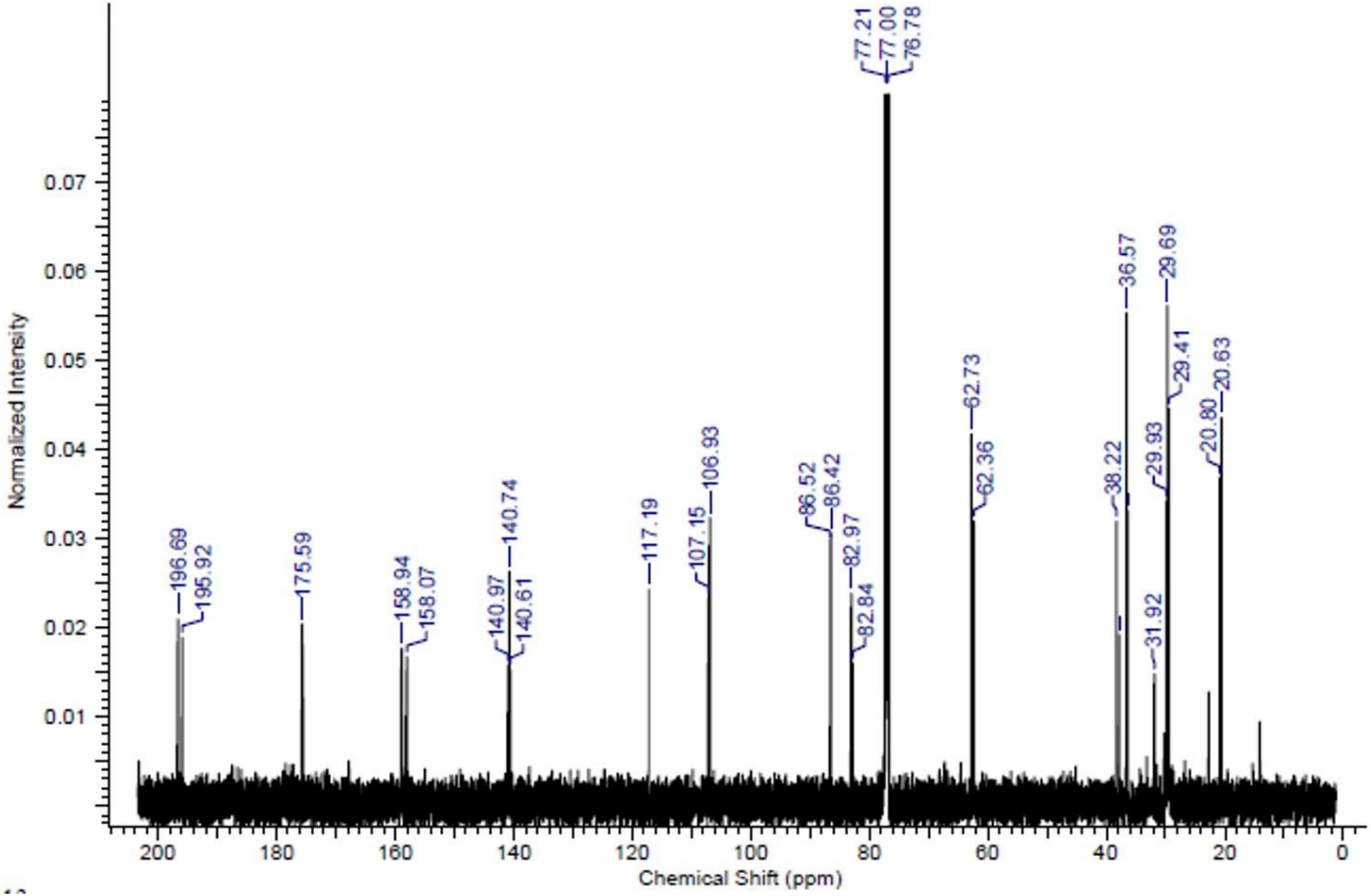

Figure S17. COSY spectrum of **16** (500 MHz, $CDCl_3$).

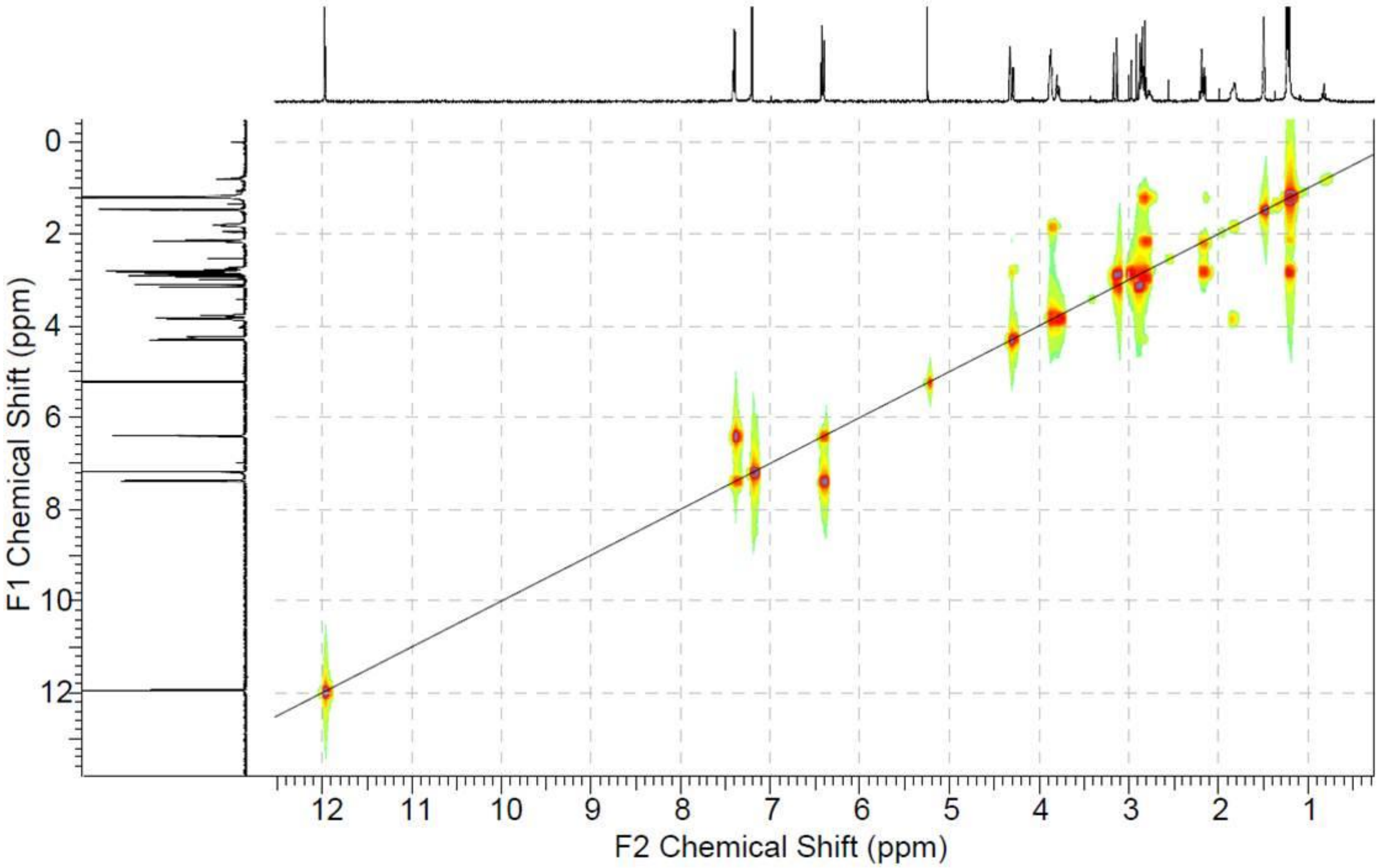

Figure S18. HSQC spectrum of **16** (500 MHz, $CDCl_3$).

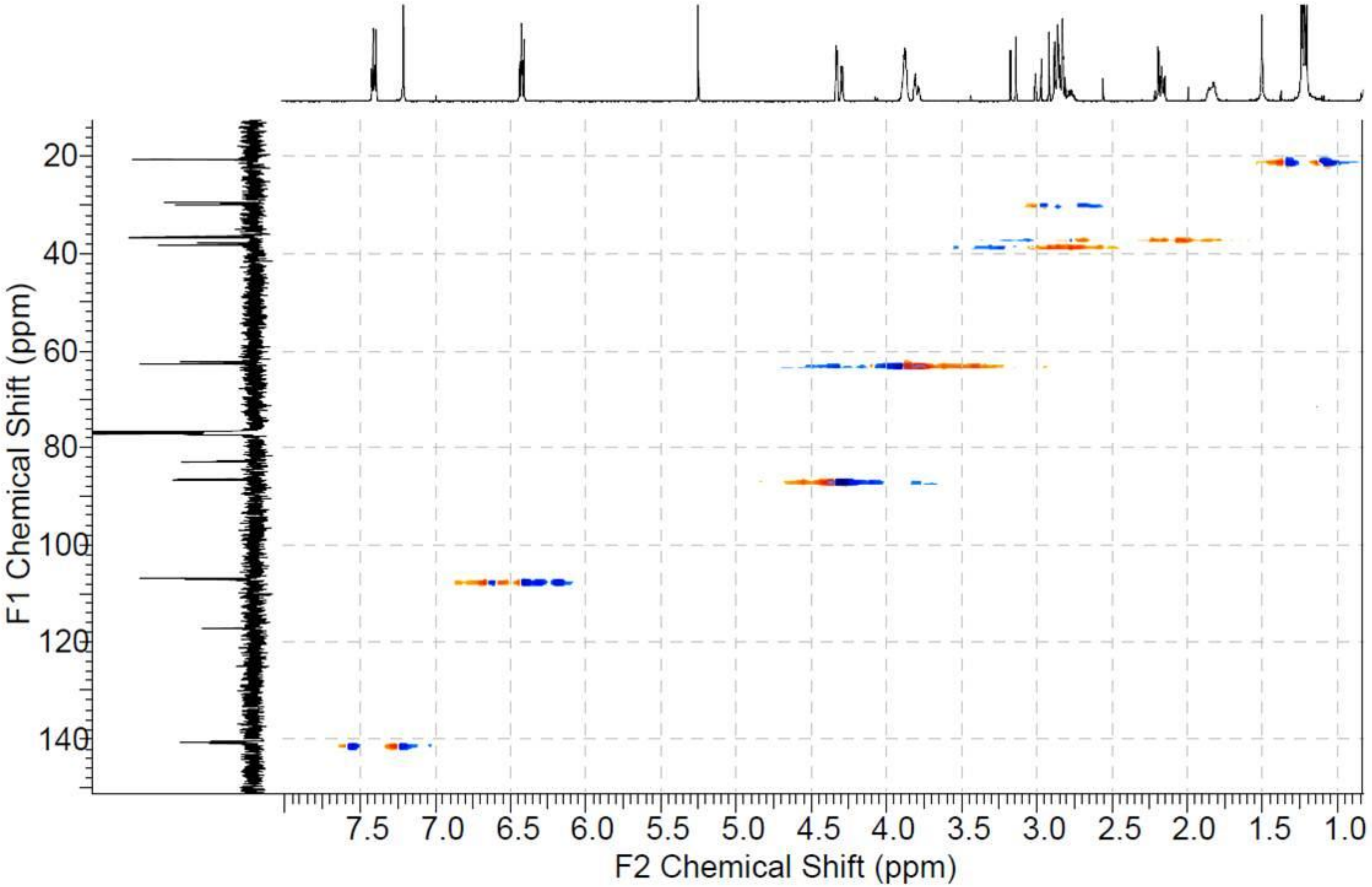

Figure S19. HMBC spectrum of **16** (500 MHz, $CDCl_3$).

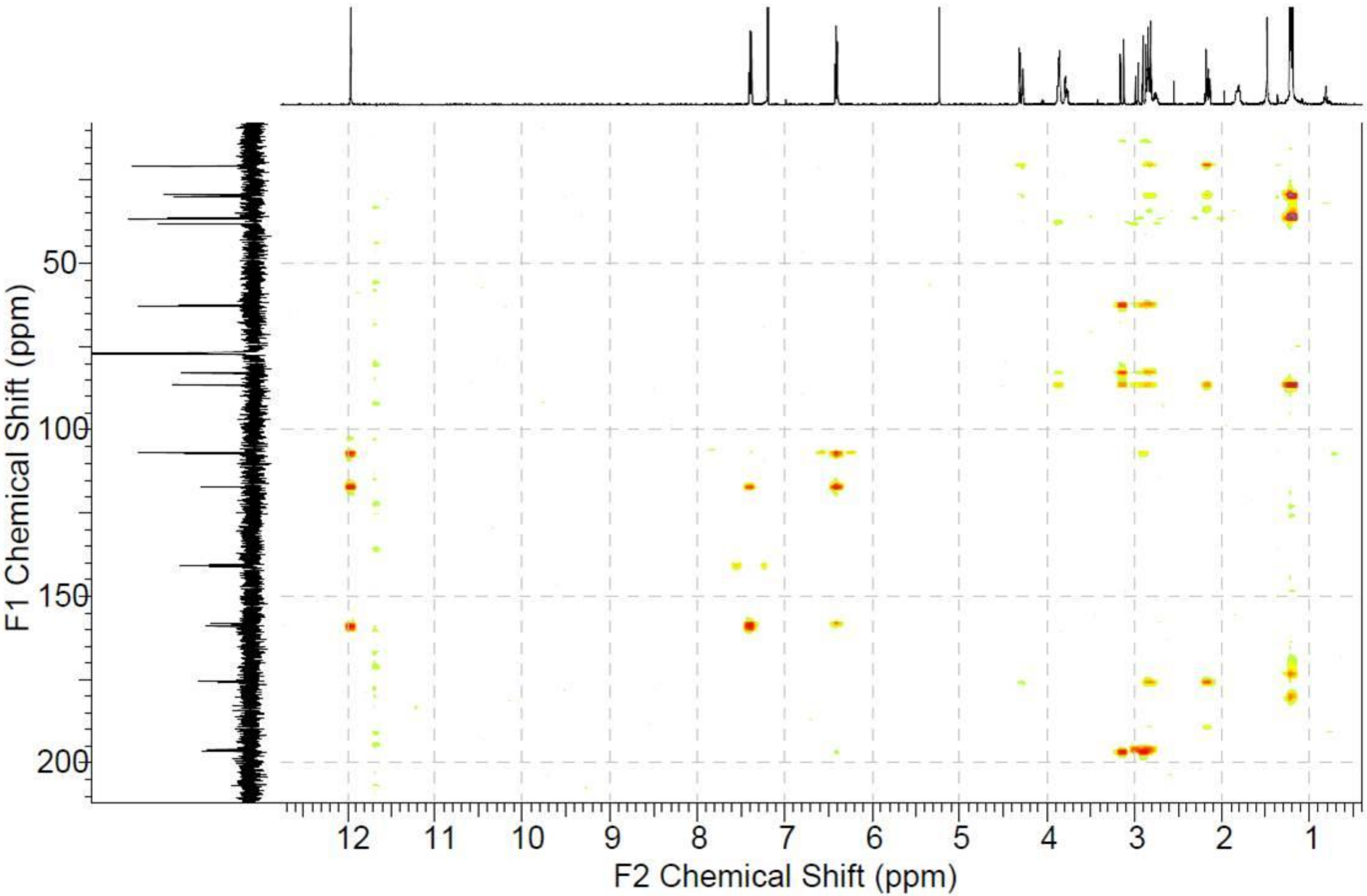

Figure S20. Expansion of ROESY spectrum of **16**, 1.0–5.0 ppm (600 MHz, $CDCl_3$).

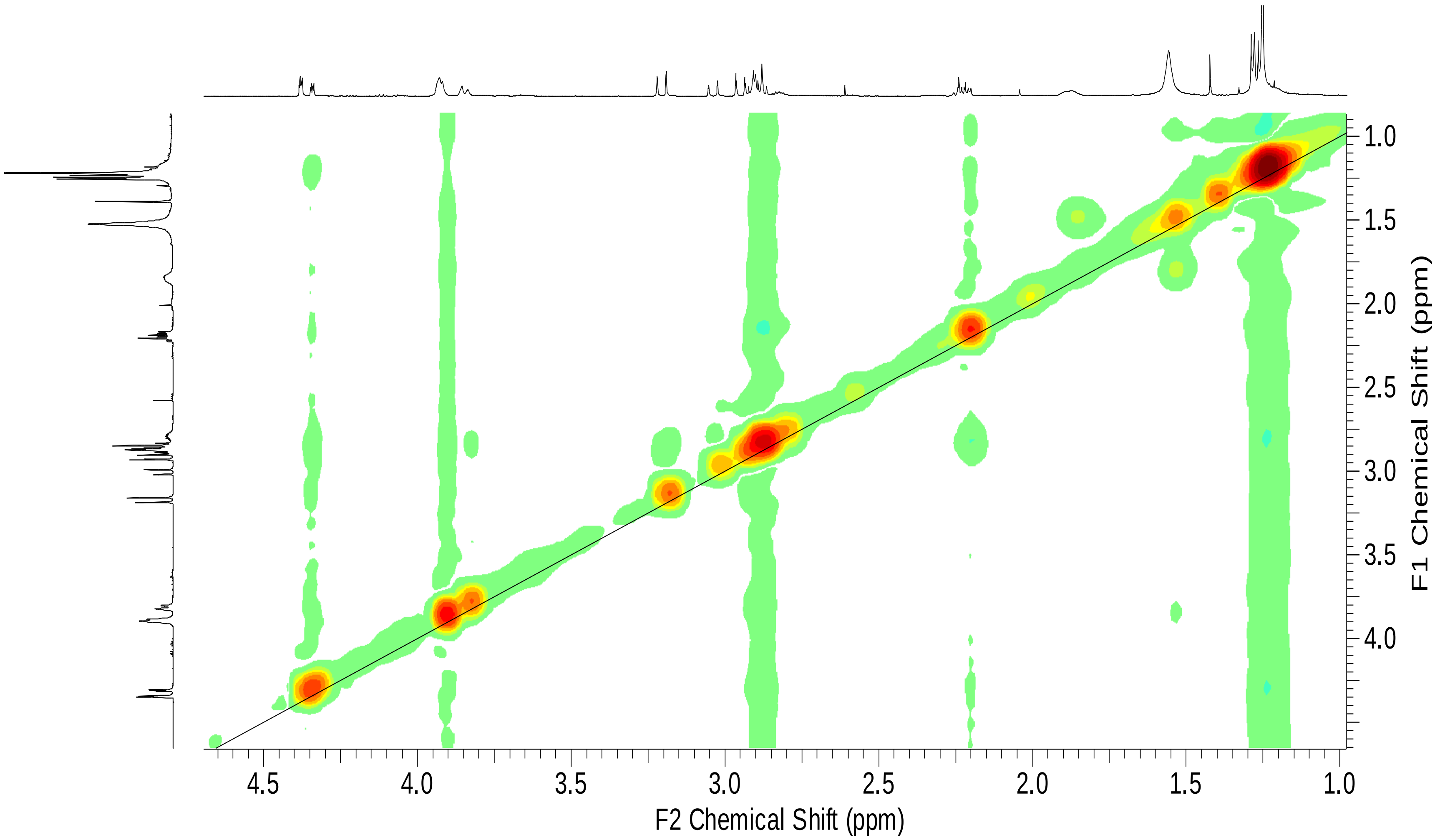

Figure S21. Isolation scheme for lipid **18**. Red highlights indicate antimalarial activity.

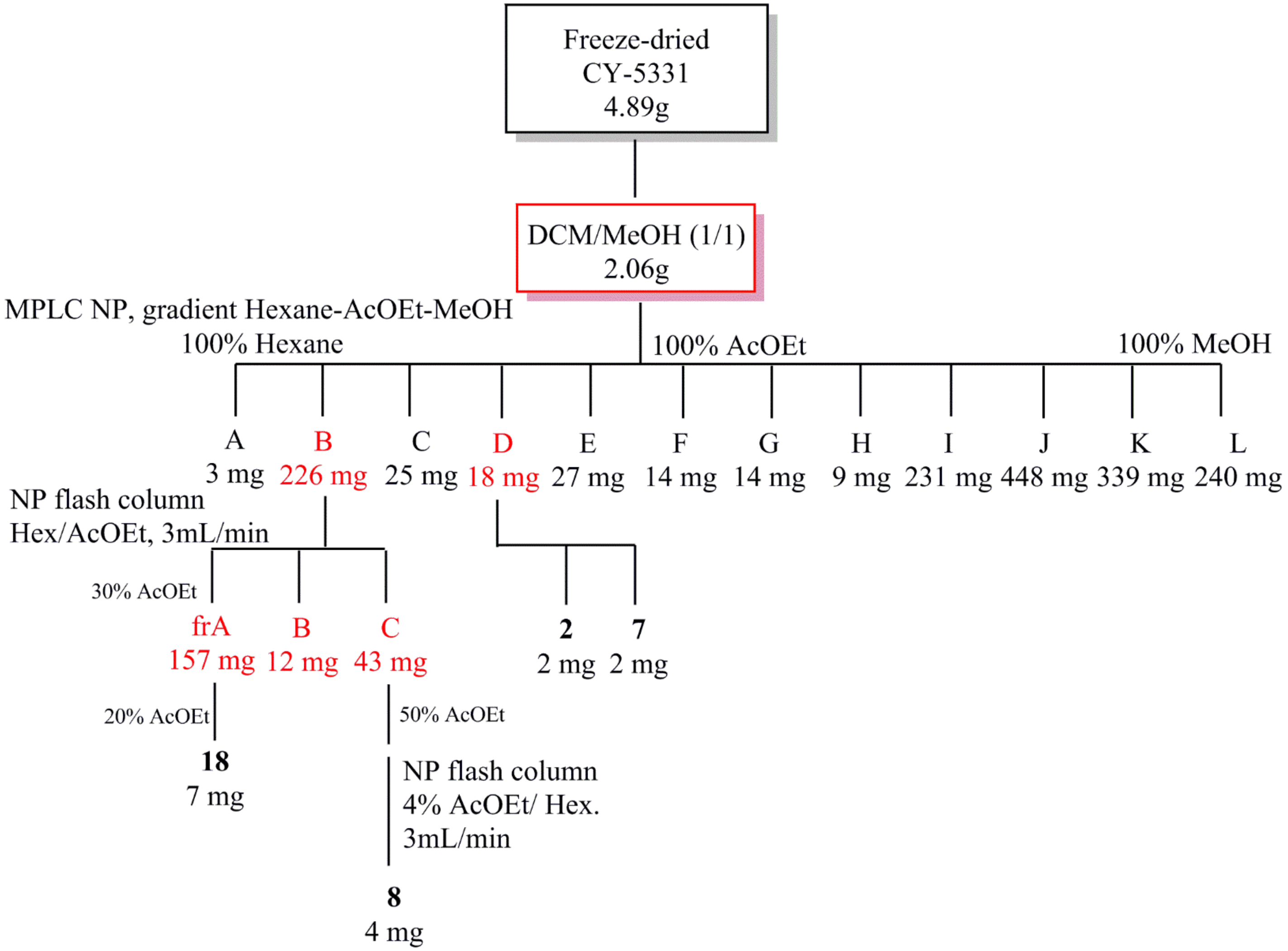

Figure S22. ^{1}H NMR spectrum of **18** (500 MHz, $CDCl_3$).

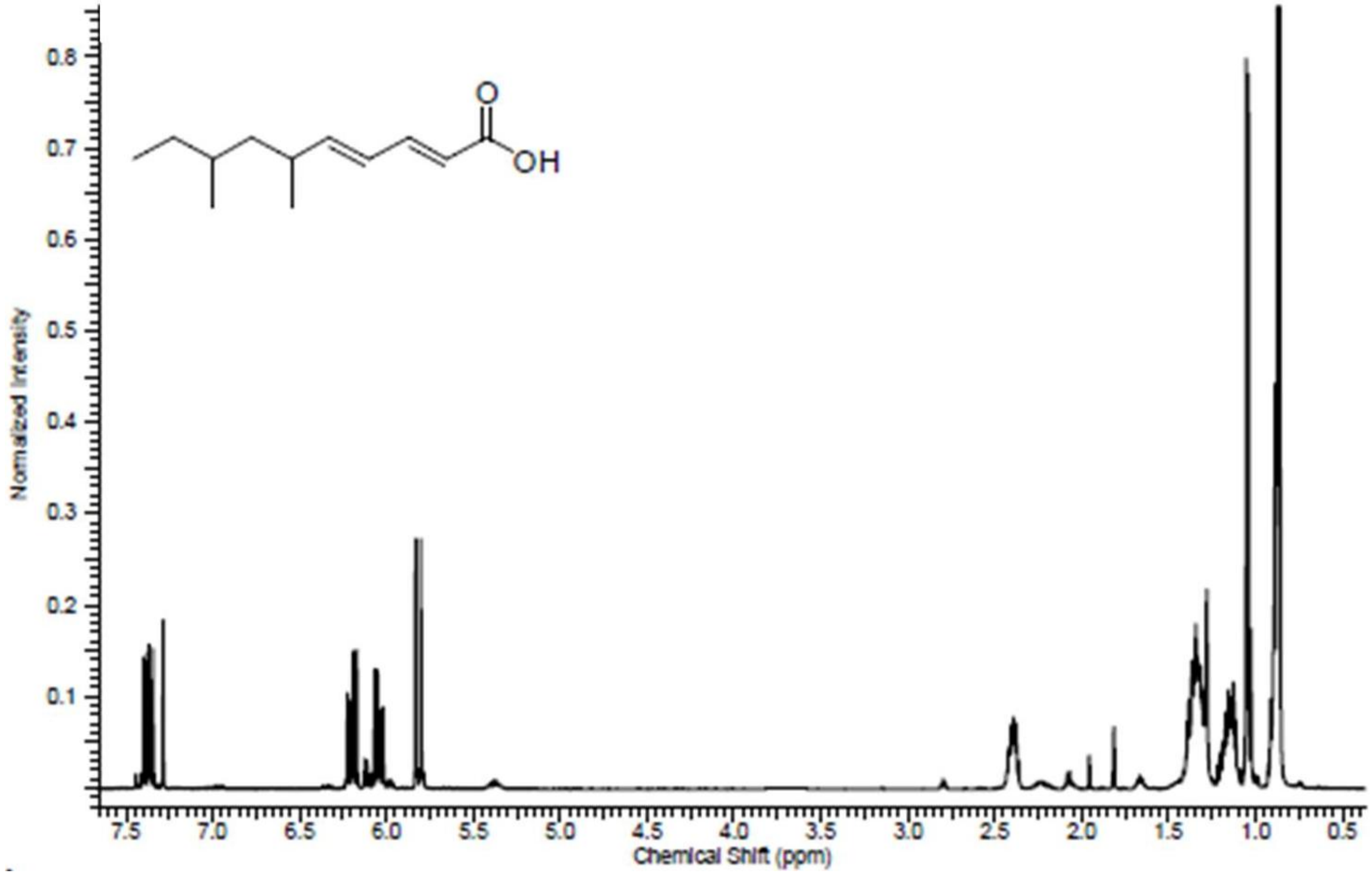

Figure S23. ^{13}C and DEPT NMR spectra of **18** (125 MHz, $CDCl_3$).

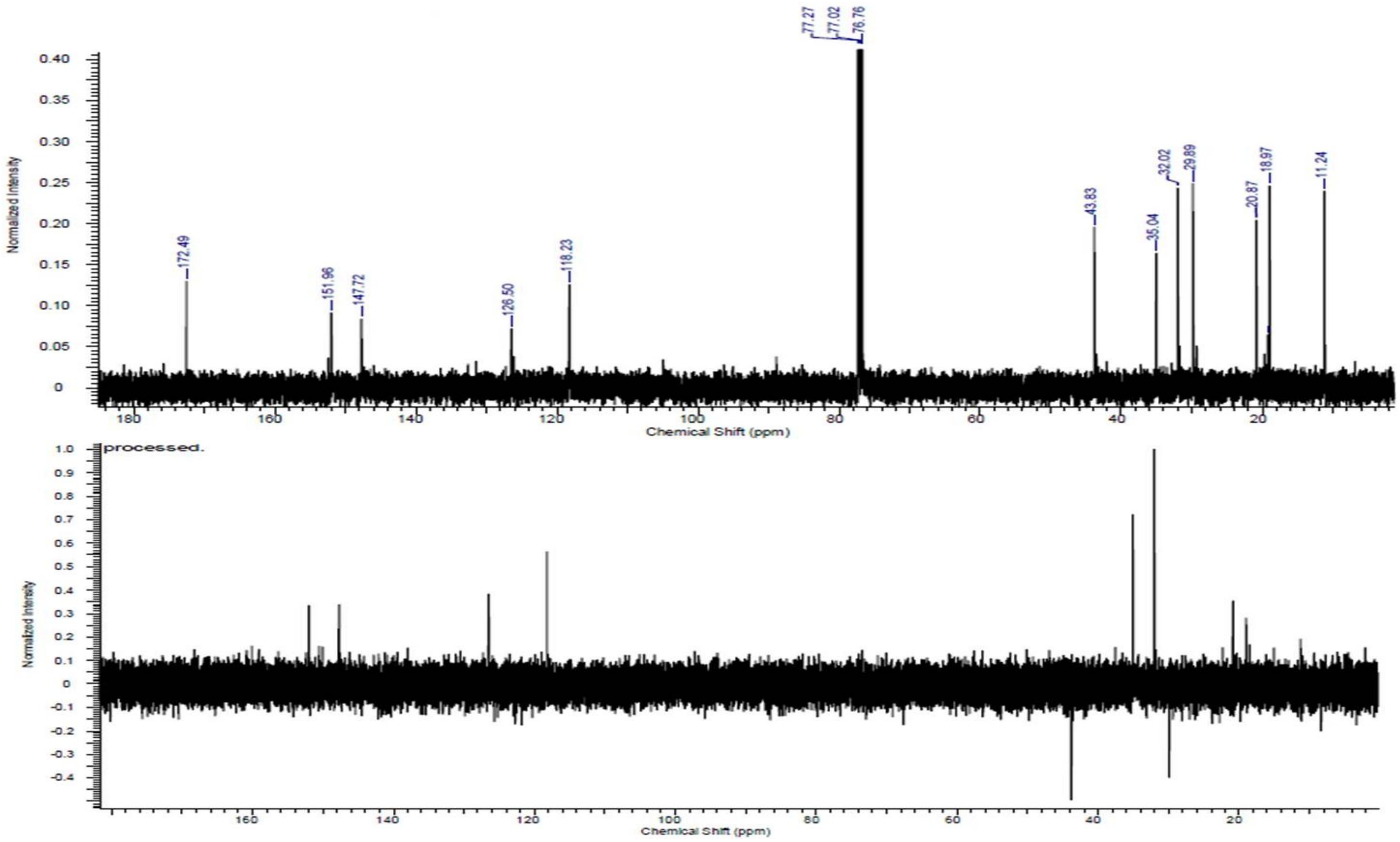

Figure S24. COSY spectrum of **18** (500 MHz, $CDCl_3$).

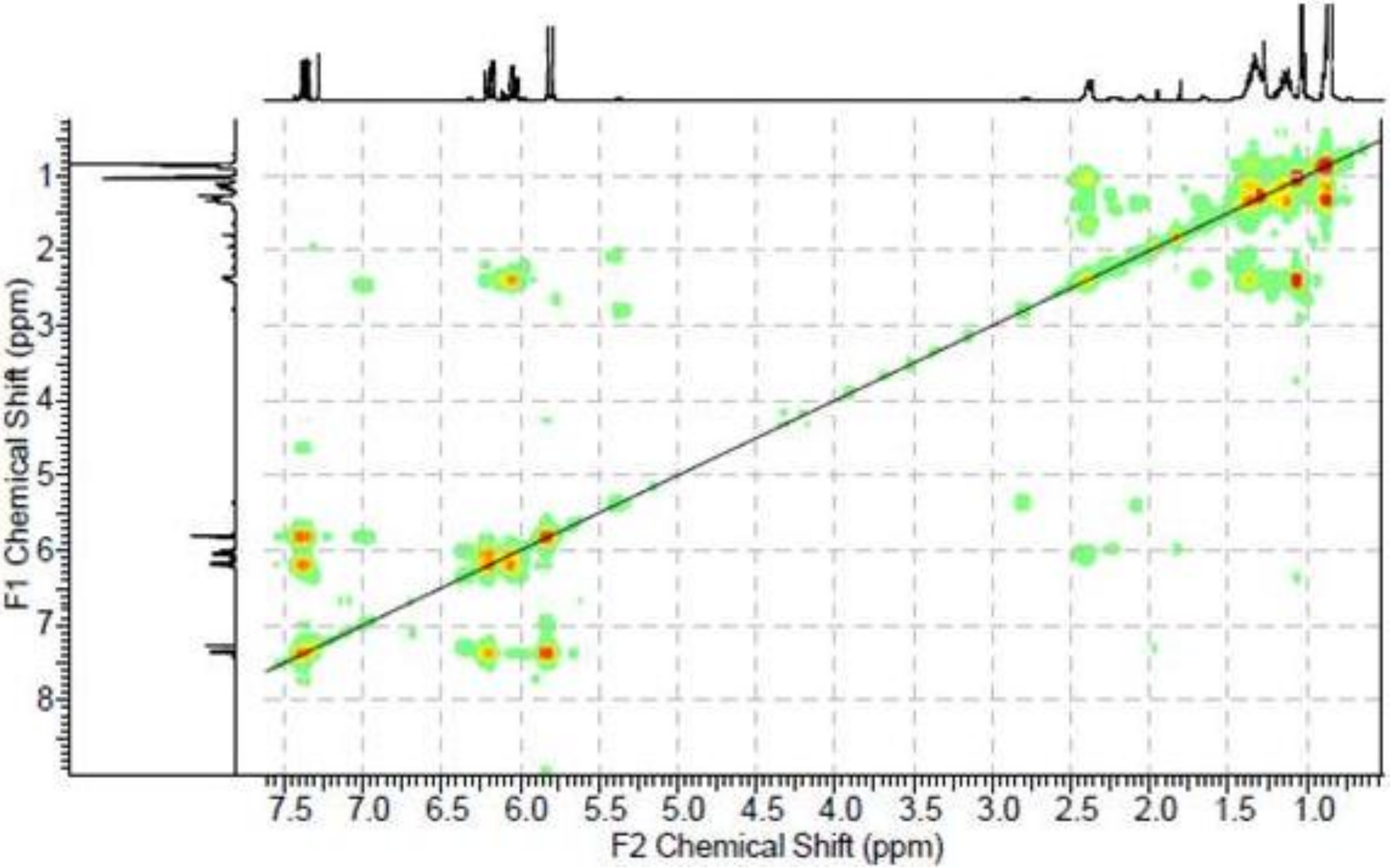

Figure S25. HSQC spectrum of **18** (500 MHz, $CDCl_3$).

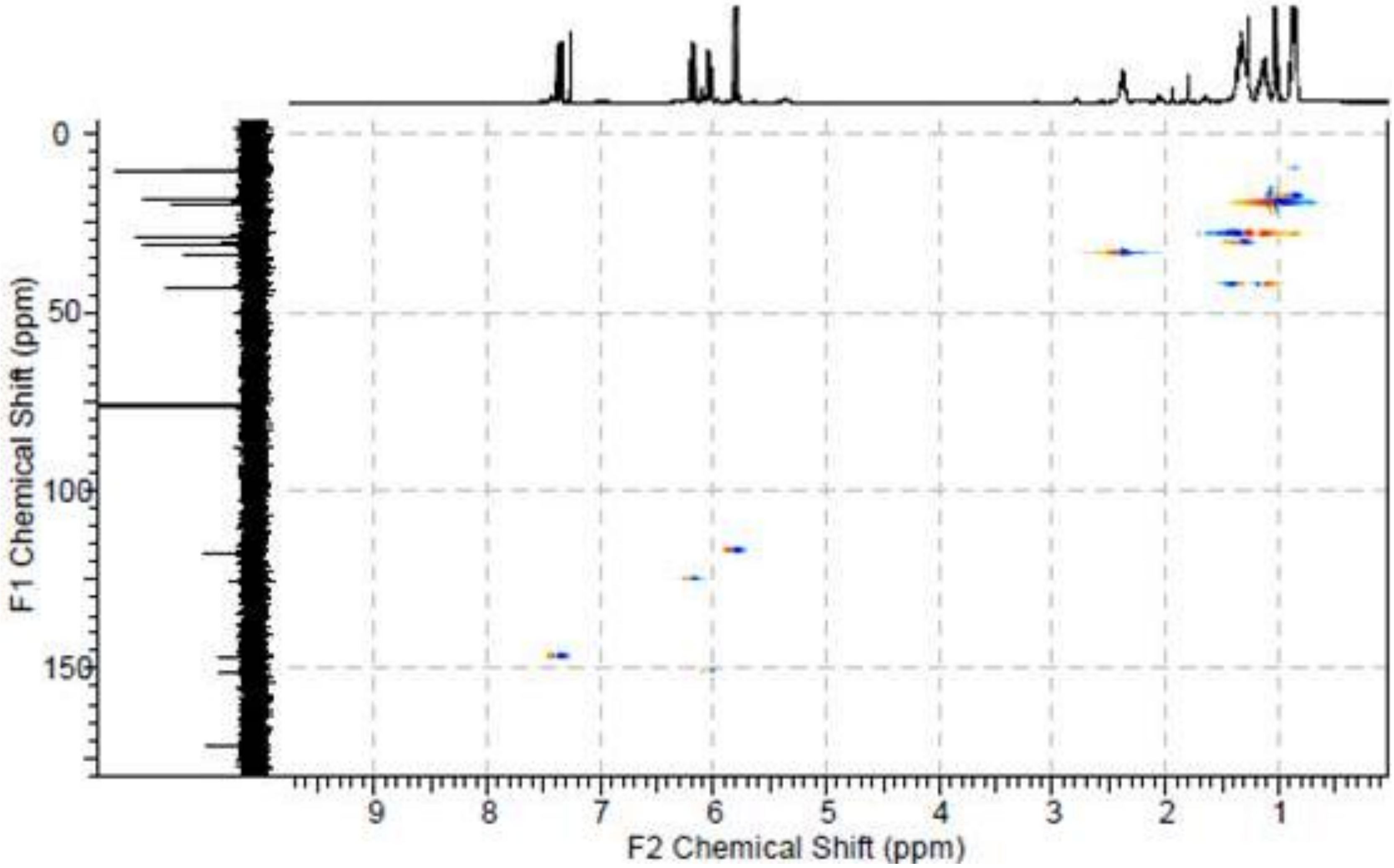

Figure S26. HMBC spectrum of **18** (500 MHz, $CDCl_3$).

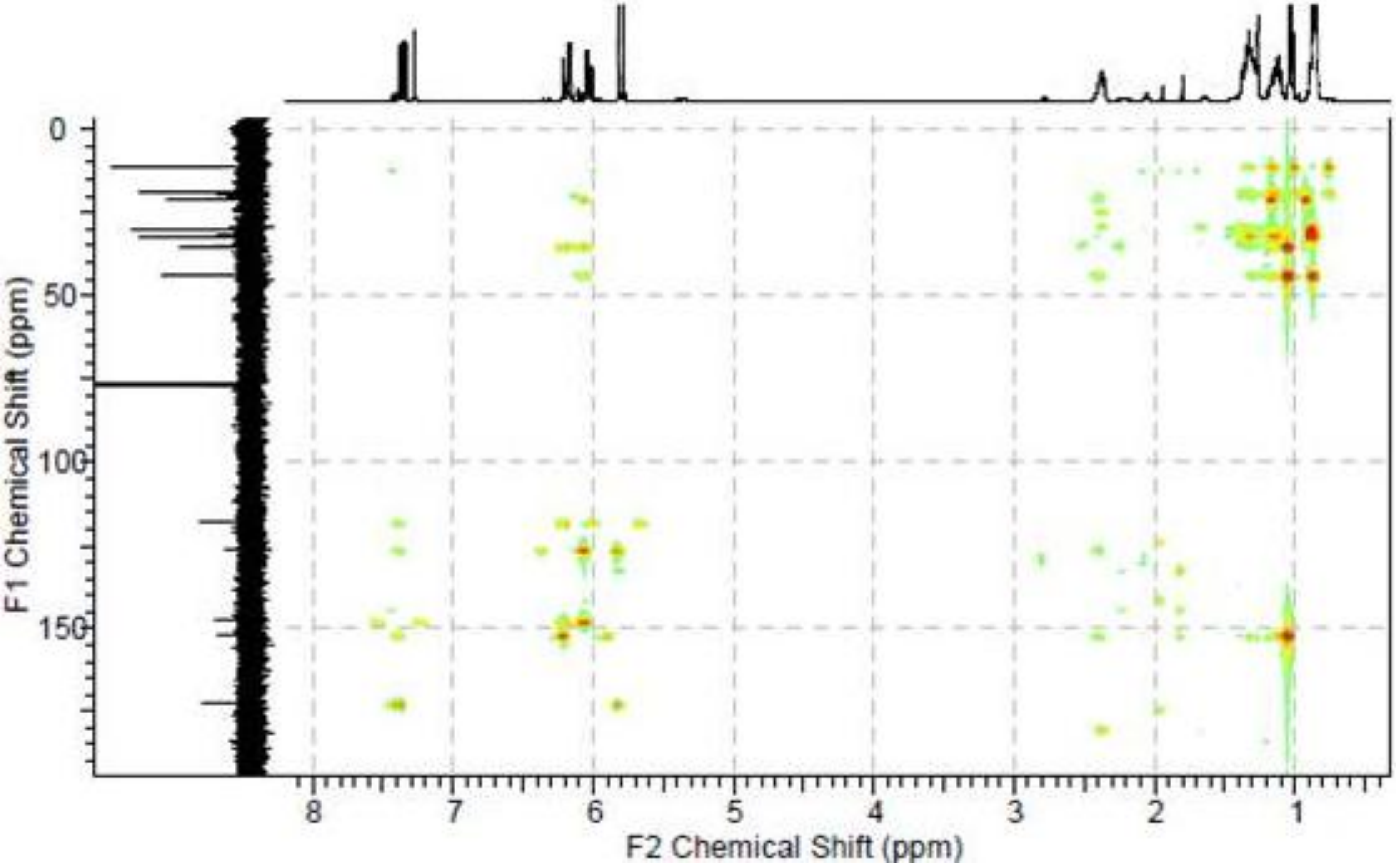

Supplement: Supplementary File 1 — Supplementary Information (PDF, 1713 KB) [file marinedrugs-11-05036-s001.pdf]
